# Development, Calibration and Performance of an HIV Transmission Model Incorporating Natural History and Behavioral Patterns: Application in South Africa

**DOI:** 10.1371/journal.pone.0098272

**Published:** 2014-05-27

**Authors:** Alethea W. McCormick, Nadia N. Abuelezam, Erin R. Rhode, Taige Hou, Rochelle P. Walensky, Pamela P. Pei, Jessica E. Becker, Madeline A. DiLorenzo, Elena Losina, Kenneth A. Freedberg, Marc Lipsitch, George R. Seage

**Affiliations:** 1 Department of Epidemiology, Harvard School of Public Health, Boston, Massachusetts, United States of America; 2 Divisions of General Medicine and Infectious Disease and the Medical Practice Evaluation Center, Massachusetts General Hospital, Boston, Massachusetts, United States of America; 3 Division of Infectious Diseases, Brigham and Women's Hospital, Boston, Massachusetts, United States of America; 4 Center for AIDS Research, Harvard University, Boston, Massachusetts, United States of America; 5 Departments of Biostatistics and Epidemiology, Boston University School of Public Health, Boston, Massachusetts, United States of America; 6 Department of Orthopedic Surgery, Brigham and Women's Hospital, Boston, Massachusetts, United States of America; 7 Department of Health Policy and Management, Harvard School of Public Health, Boston, Massachusetts, United States of America; 8 Center for Communicable Disease Dynamics and Department of Immunology and Infectious Diseases, Harvard School of Public Health, Boston, Massachusetts, United States of America; Lady Davis Institute for Medical Research, Canada

## Abstract

Understanding HIV transmission dynamics is critical to estimating the potential population-wide impact of HIV prevention and treatment interventions. We developed an individual-based simulation model of the heterosexual HIV epidemic in South Africa and linked it to the previously published Cost-Effectiveness of Preventing AIDS Complications (CEPAC) International Model, which simulates the natural history and treatment of HIV. In this new model, the CEPAC Dynamic Model (CDM), the probability of HIV transmission per sexual encounter between short-term, long-term and commercial sex worker partners depends upon the HIV RNA and disease stage of the infected partner, condom use, and the circumcision status of the uninfected male partner. We included behavioral, demographic and biological values in the CDM and calibrated to HIV prevalence in South Africa pre-antiretroviral therapy. Using a multi-step fitting procedure based on Bayesian melding methodology, we performed 264,225 simulations of the HIV epidemic in South Africa and identified 3,750 parameter sets that created an epidemic and had behavioral characteristics representative of a South African population pre-ART. Of these parameter sets, 564 contributed 90% of the likelihood weight to the fit, and closely reproduced the UNAIDS HIV prevalence curve in South Africa from 1990–2002. The calibration was sensitive to changes in the rate of formation of short-duration partnerships and to the partnership acquisition rate among high-risk individuals, both of which impacted concurrency. Runs that closely fit to historical HIV prevalence reflect diverse ranges for individual parameter values and predict a wide range of possible steady-state prevalence in the absence of interventions, illustrating the value of the calibration procedure and utility of the model for evaluating interventions. This model, which includes detailed behavioral patterns and HIV natural history, closely fits HIV prevalence estimates.

## Introduction

South Africa (SA) carries the heaviest burden of HIV disease in the world, with 6.1 million people infected and HIV prevalence of 17.9 among adults aged 15–49 [1]. Heterosexual transmission of HIV is the leading cause of new HIV infections among adults [2]. Simulation models can capture key features of HIV transmission within a population using explicit assumptions about the timing of infectiousness and the extent and distribution of sexual behavior within the population. Such models can and have been used to assess the likely impact of treatment and prevention interventions [3], [4].

HIV transmission dynamics, and the potential effect of interventions on them, are driven by the natural history of infection [5]–[7], heterogeneity in sexual behavior, assortativity of selecting sexual partners, and partnership concurrency [8]–[14]. Although a number of ongoing community-based randomized trials are assessing the impact of changing some of these factors on HIV incidence and prevalence, HIV simulation models provide an additional method to explore these questions in an effective and timely manner [3], [4]. Models also allow investigators to examine the impact of adjusting multiple interventions at once. Combining the natural history of HIV dynamics and human behavior results in complex models, and few systematic efforts have incorporated both aspects into a single transmission model [15]–[22]. Models incorporating these simultaneous processes, along with large changes in infectiousness during HIV progression in an individual, may give substantially different results from models that average over or ignore heterogeneities in sexual behavior and infectivity [6], [23], [24], in part because for HIV, changes in individual infectiousness and epidemic growth both occur over a period of several years.

For these reasons, we developed a single individual-based simulation model of HIV transmission dynamics, the CEPAC (Cost-Effectiveness of Preventing AIDS Complications) Dynamic Model (hereafter called the CDM), which links to a previously published disease model (CEPAC) and includes detailed sexual mixing among heterosexuals. This model was calibrated, using a fitting procedure based on a Bayesian Melding approach [25]–[29].

## Methods

### Model overview

**Figure 1:**
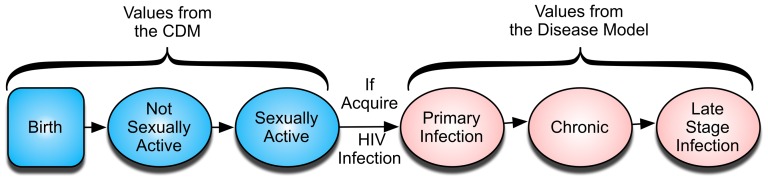
General health states. When HIV-uninfected individuals (blue) in the CDM acquire HIV (red) the health state values are gathered from the Disease Model. Individuals can die during any of the states depicted.

The CDM is a stochastic, agent-based model designed to simulate sexual transmission of HIV in a population. The agents in the CDM are males or females who become sexually-active and then form and dissolve sexual partnerships at rates dependent on their sexual risk behavior (high- or low-risk) and commercial sex worker (CSW) status. Individuals who become HIV-infected during a sexual act with an HIV-infected partner progress through stages of HIV infection via the CEPAC International Model (hereafter called the Disease Model) [30]–[35], potentially transmitting HIV to his/her partners and eventually dying of HIV infection or other causes (Figure 1). The CDM captures the key features of sexual mixing and human behavior that impact HIV transmission, and links with the Disease Model to incorporate HIV natural history (CD4 count, HIV RNA, development of opportunistic infections (OIs)), and mortality of HIV-infected and HIV-uninfected individuals. The programming platform and computational performance of the CDM is outlined in Text S1.

The Disease Model is a state-transition model of HIV natural history, which has been described elsewhere in detail [31]–[33], [35]. In brief, disease progression in the Disease Model is characterized as a sequence of monthly transitions from one “health state” to another (Figure 1). Health states are defined as follows: primary infection is the first three months post-infection, chronic infection immediately follows primary infection and continues until the individual's CD4 count drops below 50/mm^3^; late-stage infection occurs when the individual's CD4 drops below 50/mm^3^. HIV RNA is stochastically assigned by the disease model and determines the monthly decline in CD4 count, which in turn leads to increased risks of OIs and HIV-related mortality [36], [37].

The basic partnership selection framework of the CDM is that males choose females for various types of partnerships based on the difference between both partners' ages, current relationship status, and behavioral risk (high- or low-risk) (Tables 1 and S1). In the CDM, the probability that any act between an HIV-infected and uninfected partner results in transmission depends on the disease stage if the infectious partner is in primary or late-stage infection and on HIV RNA if in chronic, condom use in that sexual act, the protective efficacy of condoms, the circumcision status of the uninfected male partner, and the protective efficacy of circumcision (Text S2 and Table S1).

**Table 1:** Prior ranges and posterior weighted means for model parameters varied in calibration of a dynamic model of HIV transmission in South Africa.

Parameter Description	Prior Range for Fitting Procedure	Sources for Priors	Posterior Weighted Mean	Ratio of Variance of Priors to Variance of Posteriors
Chance of a female becoming a CSW/Proportion CSW at baseline	0.01–0.04	[91]	0.021	1.00
Proportion of males in the HR group	0.07–0.4	[92]–[96]	0.21	1.29
Proportion of non-CSW females in the HR group	0.01–0.30	[91], [94], [96]–[99]	0.16	1.05
Monthly steady partnership acquisition rate for low-risk males	0.002–0.008	*	0.004	2.31
Monthly regular partnership acquisition rate for low-risk males	0.0001–0.10	**	0.056	1.07
Monthly casual partnership acquisition rate for low-risk males	0.0001–0.15	[100]–[103]	0.058	1.95
Number of acts per month per regular partnership***	4–11	[65], [66], [99], [104]	7.46	1.00
Monthly CSW partnership acquisition rate for low-risk males	0.0001–0.10	****	0.045	1.71
High-risk multiplier	1–10	MA	5.11	1.09
High-risk Multiplier CSW	30–100	MA	72.17	1.00
Partner acquisition multiplier while in steady partnership for low-risk males	0–1	MA	0.39	1.03
Assortativeness parameter for steady, regular and casual partnerships	0.2–0.8	MA	0.51	1.02

* Calculations based on the proportion HR male, the proportion SA male in a steady partnership, the duration of a steady partnership, and the HR multiplier.

** Calculations based on the range of the partner acquisition multiplier while in steady partnership for low-risk males, the proportion in a regular partnership and the HR multiplier.

***This parameter follows a discrete uniform distribution.

**** Calculations based on proportion CSW, proportion of males who seek a CSW, proportion HR males who seek a CSW and number of acts per CSW.

HR: high-risk group, LR: low-risk group, CSW: commercial sex worker, SA: sexually-active, and MA: modeling assumption MA.

We modeled sexual acts using four types of partnerships: 1) A steady partnership (similar to a marriage), defined as a main partnership of long duration (Table S1) formed between a sexually-active, single male and a sexually-active, single, non-CSW female; 2) A regular partnership, defined as an ongoing partnership of shorter duration (less than the duration of a steady partnership, Table S1) formed between a sexually-active male and a sexually-active, non-CSW female; 3) A casual partnership, defined as one sexual act by a sexually-active male and sexually-active, non-CSW female; and 4) A CSW partnership, defined as one sexual act between a sexually-active male and CSW female assumed to involve payment or exchange of goods or services for sexual acts.

### Dynamic Model Steps

#### Model initialization

We used the CDM to generate an initial population, one individual at a time, using specified values for population size, proportion male, proportion of males circumcised, age of sexual debut, and proportion of females who are CSW. The initial population was divided into nine age strata based on user-specified proportions of the population in each age range. Individuals younger than the user-defined age of first sex for the CDM were classified as non-sexually-active. With fixed sex- and CSW status-specific probabilities, sexually-active individuals were assigned to be high-risk; otherwise they were low-risk. A complete list of the CDM parameters used to describe population characteristics, sexual partnership characteristics, partnership selection, and probability of transmission is provided in Tables 1 and S1. In the CDM, each sexually-active male has a mean partner acquisition rate for each partnership type. We allowed the monthly mean partner acquisition rates for steady, regular and casual partnerships to differ for high-risk males compared to low-risk males by a high-risk multiplier, a parameter multiplied by each of the four low-risk partner acquisition rates. Similarly, we applied a CSW multiplier for high-risk compared to low-risk males to the partner acquisition rate for CSW partnerships.

We ran the CDM for an initialization period of 50 years (roughly 5 times the duration of a steady partnership in the model) in the absence of HIV prior to time 0, so that the steady and regular partnerships form and dissolve over the average lifespan of individuals in the model, allowing for the distribution of these partnerships to approach equilibrium. The formation of these partnerships is described below (see *Partnership updates*). After the 50-year initialization period, hereafter referred to as time 0, the CDM is seeded with HIV-infected individuals with a specified age, gender, and CSW status (Table S1). The Disease Model assigns these individuals an initial CD4 and HIV RNA.

#### Births

Each month, after deaths are tallied in the model, the CDM generates births based on the population size and the average birthrates for SA from 1985–2002 [38].

#### Monthly updates for individuals

Every month, the model increases each individual's age by one month. When an individual reaches the age of sexual debut, age 17 (Table S1) [39]–[41], he/she moves into the sexually-active, single pool and is classified as low- or high-risk based on the gender- and CSW status-specific probabilities used in initialization (Table 1). Individuals remain in their assigned HIV-risk group or CSW for their lifetime. Newly sexually-active males draw a monthly mean partner acquisition rate for each of the four partnership types based on their risk group (see *Model initialization*). All newly sexually-active females have a defined probability of becoming a CSW.

The health status of prevalent HIV-infected individuals in the CDM is updated every month with data gathered from the Disease Model. Similarly, newly HIV-infected individuals acquire their health status from the Disease Model beginning at the time of infection. Death of HIV-infected and uninfected individuals depends on age, gender and the probability of AIDS- and non-AIDS-related causes of death [37], [42]–[44]. Upon death, the individual is removed from the active dynamic population and the individual's sexual partnerships end.

#### Partnership Updates

Partnerships are updated every month allowing new partnerships to be formed and ongoing partnerships to continue or dissolve, depending on the duration of the given partnership type (Table S1). For each male, the number of sexual acts and probability of condom use that month is updated for each partnership type (Table S1).

For each partnership formed, the male selects a female partner based on her partnership (single or non-single) and CSW status applying the user-defined partner selection weights associated with each partnership type (Table S1). Eligible age categories for the female partners are based on the user-defined average age difference for that partnership type. For steady, regular and casual partnerships, once the age category has been selected, the male selects the risk group (high or low) from which his partner is drawn, based on the value of assortativeness (*assort*) parameter. A male selects his partner randomly from his own risk group with a probability of *assort*, and randomly from either risk group with a probability of 1-*assort*. All females in the selected partnership status, risk group, and age category have an equal probability of being chosen by the male. The female selected by the male forms a partnership of a defined type with that male. If a steady or regular partnership is formed, the duration of that partnership is drawn from a specified range for that partnership type (Table S1). If a steady partnership is formed, the partnership status for each partner is changed to non-single and the individuals cannot form any other steady partnerships until the given steady partnership has ended. The CDM tracks the type and number of partnerships each individual forms each month.

The CDM allows males to engage in multiple partnerships within a given month. However, males in steady partnerships may have a decrease in their partner acquisition rates for forming other partnerships, determined by a partner acquisition multiplier (Table 1). We also allow females in the CDM to engage in multiple partnerships within a month by using the partnership selection weights (Table S1). These weights define the probability that a male will select a single or non-single female to form a steady, regular or casual partnership. For example, these weights dictate that a male who is to form a regular partnership has a higher chance of selecting a single female than of selecting a non-single female. Also, in general, females in a steady partnership have a decreased probability of being selected compared to single females, and males can only select a CSW to engage in a CSW partnership. To reflect the decrease in sexual activity as individuals age, the partnership acquisition rates and sexual acts are adjusted by a yearly age discounting factor, starting at the age of 50 (Table S1).

In the model, once all males have formed all new partnerships for the given month, all sexual acts between partners occur for that month and the model determines whether HIV transmission has occurred for each sexual act within each partnership (Text S2). For each incident HIV infection, the initial HIV RNA and CD4 count is obtained from the Disease Model using distributions for incident cases. After all of the monthly transmission outcomes have been determined, partnerships that have reached the end of their duration, including any casual or CSW partnerships that were formed in the same month, are ended. Upon dissolution of a steady partnership, the individuals' partnership status changes back to single.

### Internal validity of the Dynamic Model

Internal validation of the CDM was performed in a three step process to evaluate the model's structure and performance. First, we evaluated the internal validity of the CDM by examining randomly selected detailed reports or traces, which provide monthly information on each partnership formed, the number of acts per partnership, the health status and demographic characteristics of the individual, and the number of new transmissions for HIV-infected individuals.

Second, the HIV transmission dynamics were validated by assessing the number of exposures that led to infections, stratified by the HIV RNA of the infector. This was done by tallying the number of unprotected sexual acts between an infected individual and their uninfected partner (exposures) and the number of infections, both stratified by the HIV RNA of the infector. We were most interested in understanding the rate of transmission for primary stage, late stage, very low viral load (HIV RNA<500 copies/ml) and chronic infection with HIV RNA>100,000 copies/ml.

Third, we examined the influence of each parameter to determine if it had the expected impact on various model outputs, such as the number of partners formed in a month, number of new infections in a month, number of deaths, etc. We conducted extreme sensitivity analyses to confirm consistent model behavior. For example, we set the probability of transmission to 0 for all HIV RNA strata and examined the model output to ensure that no HIV transmission had taken place. We then set the probability of transmission to 1 and confirmed that the model output showed that all sexual acts between discordant partners resulted in HIV transmission.

### Choice of parameter values and ranges for a model of South Africa

The parameter values related to disease progression for the South African HIV epidemic have been published previously [31], [45]. For incident HIV cases, we reviewed the literature and found the CD4 count of HIV-uninfected individuals ranged from 503 to 2051 cells/mm^3^ [46]–[62], consistent with the mean CD4 count of 890 cells/mm^3^ found by Phair [61] and 884 cells/mm^3^ used by Granich [46], [63]. We chose to use the value of 884 cells/mm^3^ as the initial CD4 count of incident HIV cases in the CDM based on additional sensitivity analyses that analyzed the impact of this parameter on life expectancy (See Text S3). The HIV RNA is >100,000 copies/ml for the first three months post-infection. The CD4 decline and distribution of HIV RNA after the first three months is outlined in Table S2. Monthly mortality probabilities are dependent on sex, age, the presence of acute and history of OIs, and CD4 count [37], [64]. Thus, the model includes both AIDS and non-AIDS related causes of death.

Demographic and behavioral parameters representative of the pre-antiretroviral therapy (ART) HIV epidemic in South Africa starting in 1990 were obtained from the literature (Tables 1 and S1). The data gathered for the input parameter values were selected based on the study design, sample size and year the study was performed. Sixty-four of the 76 behavioral parameters of the model were given fixed values; these mainly represent selected demographic parameters and the HIV RNA-dependent probability of transmission including the initial age distribution across the nine age strata and the initial number of incident HIV cases stratified by age and gender (Table S1). The probability of transmission per act was fixed at values ranging 0.0001 to 0.0023 for different ranges of HIV RNA during chronic infection, with unique probabilities for primary (0.0082) and late-stage infection (0.0036) (Table S1) [65]–[68].

After a complete literature search was conducted to gather the most robust estimates for the behavioral parameters, there were 12 parameters in the model for which there were no or few direct measurements in the literature (Table 1). Therefore, the values for these 12 remaining parameters were varied across simulations in the calibration exercises (see *Dynamic Model Calibration* below). These parameters included, but are not limited to, the proportions of individuals in different risk groups, partner acquisition rates in each risk group for all partnership types, and the assortativeness of mixing.

### Dynamic Model Calibration

In brief, the CDM transforms the input, consisting of multiple behavioral, disease-related, and demographic input parameters (Tables 1, S1, and S2), into the output, HIV prevalence over a given time period. We used a Bayesian Melding-like procedure [25]–[28], [69] to take prior information on the input parameters and simulated behavioral properties of the population, then use priors on the behavioral and epidemiologic outputs to exclude parameter sets and combined it with a measure of how well the model output (HIV prevalence) of each particular parameter value combination fit annual estimates of HIV prevalence produced by UNAIDS (Table S3) [1]. We began from a broad, multivariate uniform distribution of values for 12 behavioral and demographic parameters, which governed the sexual network formed by the entire male and female sexually-active population (Table 1). While many of the partnership formation rates and the proportions of high- and low-risk individuals are not well-constrained by available data, there are better data available for values of composite quantities that depend on them, such as the proportion of adults of each sex who are in various types of partnerships (Table S4). Therefore, we performed a three-step calibration procedure.

We began the calibration procedure producing a total of 264,225 parameter sets by randomly selecting values for each of the 12 parameters from their respective prior uniform distributions (Table 1). We ran the CDM once for each of the 264,225 different input parameter sets, which consisted of both fixed and randomly selected parameters. We then subjected the CDM output from each parameter set to three phases of calibration (Text S4). During Phase 1, we assigned likelihood weights of zero to parameter sets for which the estimated sexual partnership prevalence and sexual acts fell outside the pre-specified limits (Text S4.1 Phase 1, Table S4). Formally, this is equivalent to having a multivariate uniform prior on the composite quantities, as these limits reflected prior information on behavioral quantities that are determined by complex interactions of the partnership formation and dissolution parameters that we specified in prior distributions (Table 1). The Bayesian melding-like approach provides a rigorous framework for reconciling the prior on the parameters with the prior on composites of them, treating them as two sources of information about the parameter values.

During Phase 2 of the calibration procedure, we assessed the goodness of fit of the modeled HIV prevalence to annual prevalence estimates from UNAIDS for the parameter sets that remained with nonzero weight after Phase 1 (Text S4.2 Phase 2, Table S3). We assigned each parameter set a weight proportional to the pseudo-likelihood (Text S.4.3) that the parameter values could produce the pre-ART HIV prevalence curve defined by the UNAIDS estimates of HIV prevalence in South Africa [1], [25]–[28], [70]. Parameter sets that failed to generate an epidemic, failed to reach a prevalence <0.005 after 38 years, were given zero weights (0.005 was the prevalence in 1990 from the UNAIDS data).

During Phase 3 of the calibration procedure, we filtered the parameter sets further to ensure face validity by conformance to specified stratum-specific HIV incidence and prevalence, and CSW behavioral parameters (Table S5) at the end of the simulation (2002) (Text S4.3 Phase 3). Parameter sets failing this phase had their weights set to zero. This calibration method was a principled way to assign weights to combinations of parameter values so that they are individually consistent with measurements in the literature *and* that in combination produce modeled HIV prevalence consistent with existing data. This method conceptually combines random sampling methodologies (e.g. Latin Hypercube sampling), focused on model inputs [71], with curve-fitting and likelihood methodologies that focus on identifying parameter values that produce well-fitting outputs [29], [72]–[74].

### Post-Calibration Analyses

To assess the impact of the 12 parameters varied in the analysis on the HIV prevalence in each year from 1990–2002, we calculated partial rank correlation coefficients (PRCC) for all 12 parameters with the predicted HIV prevalence at each year (Text S5) [71]. Additionally, we generated two-dimensional heat plots showing the location of high-weighted vs. low-weighted parameter sets across each of the 132 ordered pairs of parameters (12*11), to visually identify pairwise correlations between values of parameters that produced well-fitting runs (Text S6). Histogram plots of the weighted posterior distributions for the 12 parameters were created to determine how the calibration restrictions influenced the parameter sets that passed the calibration checks (Text S.6). To provide a measure of how informative the calibration procedure was for narrowing the prior ranges, we compared the posterior means for each of the 12 parameters to its respective prior mean and calculated the ratio between the variance of its prior distribution and the variance of its posterior distribution.

## Results

### Model Validation Results

**Figure 2:**
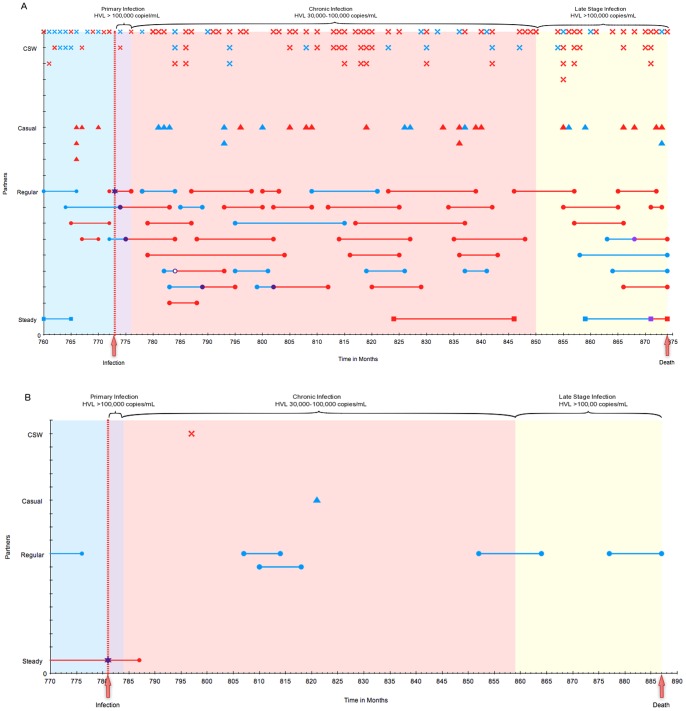
Trace plot of a selected high-risk and low-risk male. In this figure, each month is represented on the horizontal axis with multiple partners per month plotted vertically. Partnership types are represented as follows: ▪ for steady partnerships, • for regular partnerships, ▴ for casual partnerships, and **X** for CSW partnership. A blue symbol represents an HIV-uninfected female partner, a purple symbol a female partner that becomes HIV-infected that month and a red symbol a female partner who is previously HIV-infected. The duration of steady and regular partnerships are represented by a line; if that partner acquires HIV during the partnership, the time of HIV acquisition is represented by a purple symbol and a change in color of the line from green to red. The stage of HIV disease is depicted by the graph fill color, with green representing HIV-uninfected, purple primary infection, red chronic infection and yellow late-stage infection. This male depicted in Panel A is a typical high-risk male and in Panel B a typical low-risk male.

We examined randomly selected traces of individuals to evaluate the internal consistency of the CDM. All the individuals evaluated formed partnerships, had sexual acts, had updated health status, aged, and died as defined and governed by the inputs and design of the CDM. Figure 2 illustrates a trace of a high-risk (Figure 2A) and low-risk (Figure 2B) male from just prior to infection until death. These traces provide detail on all sexual partnerships and potential transmissions due to partnerships with the selected male using the best-fitting parameter set (see below).

The plot of the high-risk male (Figure 2A), shows the partnerships that individual had each month from roughly one year prior to infection until his death at age 37 (8.4 years after infection). Throughout the course of his entire life, this male formed 335 CSW, 17 casual, 65 regular and 3 steady partnerships. A regular partner infected this male (represented by a large purple star) when he was 29 years of age. After infection (vertical red line), this male infected two regular partners during his acute infection, two regular partners during chronic infection and one steady and one regular partner during late-stage infection (represented by the purple symbols). This male's behavior is consistent with model input parameters and the literature for high-risk males (Table 1).

The plot of the low-risk male (Figure 2B) shows this male's partnerships from roughly one year prior to infection by a steady partner at 32 years of age until death at the age of 41, after being infected with HIV for 9 years. This male did not infect any partners after his own infection. Throughout the course of his entire life, he formed 3 CSW, 4 casual, 10 regular and 1 steady partnerships.

To further examine the connection between the disease stage and HIV RNA strata and the probability of transmission per sexual act, we examined model output to determine the percentage of acts that resulted in transmission to an uninfected partner. These percentages were stratified by the HIV RNA bucket or disease status (primary or late-stage infection) of the HIV infected partner. A total of 0.76% of all sexual acts with an HIV-infected partner in primary stage resulted in infection, 0.24% of acts with an HIV-infected partner who had an HIV RNA >100,000 copies/ml in chronic stage infection resulted in infection, and 0.36% of acts with an HIV-infected partner with late-stage infection resulted in infection. Only 0.01% of acts with an HIV-infected individual with HIV RNA<500 copies/ml resulted in transmission to an uninfected partner. These numbers closely approximate the transmission probabilities designated in the model input, as expected (Table S1).

### Calibration results

**Figure 3:**
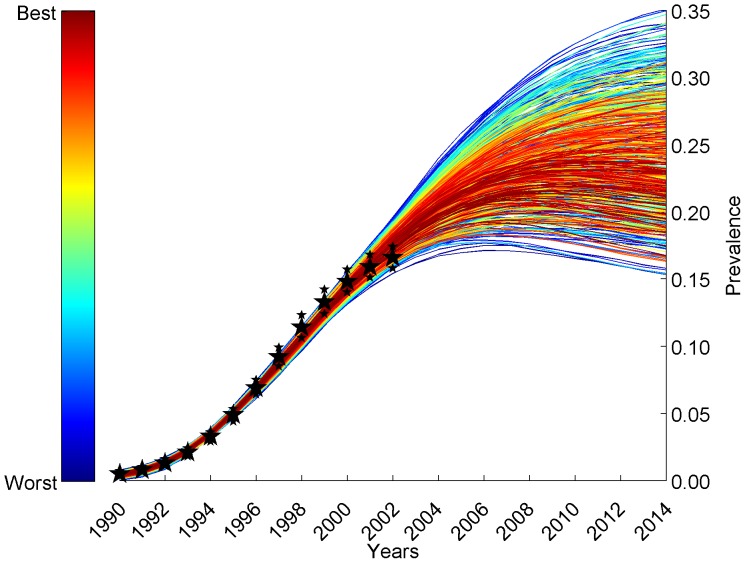
HIV epidemic curves from the fitting procedures. This graph depicts that HIV epidemic curves generated by the CDM, from the parameter sets that contribute the top 90% of the likelihood weight, closely represent the HIV prevalence in South Africa from 1990–2002 (large black stars, Table S3) [1], [70]. The estimated ranges for the South African prevalence from 1990–2002 for the UNAIDS data were plotted using small black stars.

Of the original randomly selected 264,225 parameter sets, 32,769 runs (12.4%) passed Phase 1, 29,544 parameter sets (11.2%) passed Phase 2, and 3,750 parameter sets (1.4%) passed Phase 3 calibration (Figure S1). Likelihood weights ranging from near 0 to 0.006 were assigned to each of the 3,750 simulations based on its predicted HIV prevalence's pseudo-likelihood of fit to the UNAIDS HIV prevalence in South Africa from 1990–2002 [1], [70]. The parameter sets that contributed to the top 90% of the likelihood weight were selected from these 3,750 simulations. These 564 runs closely follow the prevalence values from 1990–2002 from South Africa (Figure 3) [1], [70] and then take various trajectories after 2002. A cumulative distribution of the weights assigned to all parameter sets is presented in Figure S2; the curve with sigma  = 1 corresponds to the analyses described here in the main text.

The posterior distributions for the 12 variables varied in the fitting procedures are summarized in Table 1. The posterior mean value for most parameters was close to its respective prior mean. While the posterior variance was reduced for all parameters, the ratio between the variance of the prior and the variance of the posterior for the partnership acquisition rates and the proportion of the population defined to be high risk were the largest, indicating the strongest constraints imposed by the fit to data (Table 1). This indicates that these parameter values were most constrained by this fitting procedure relative to their prior distributions.

**Figure 4:**
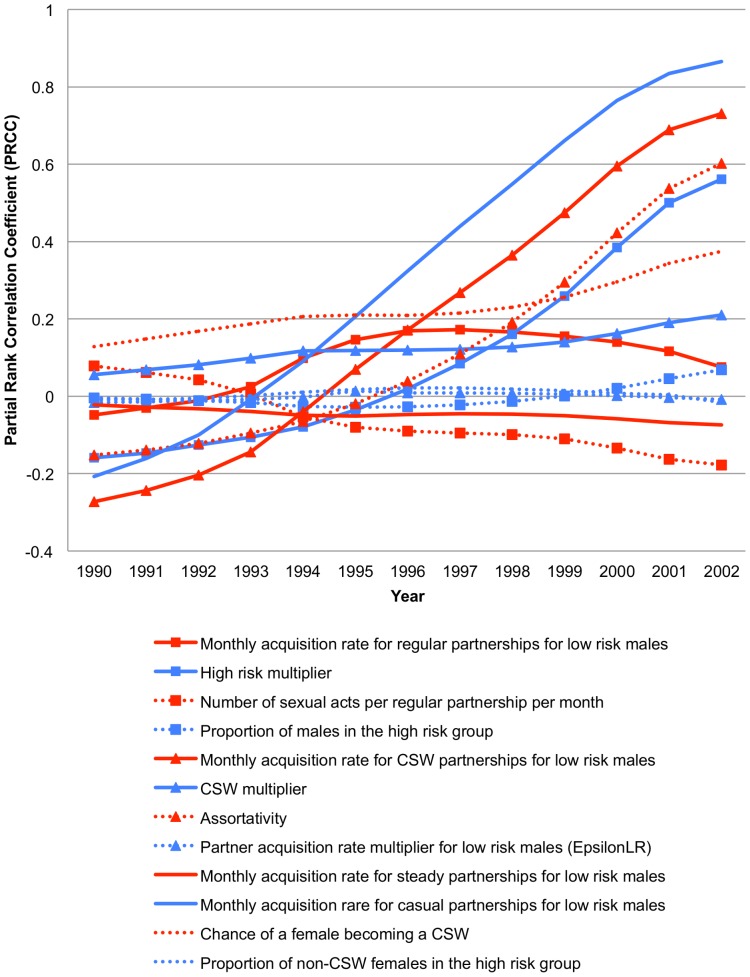
Partial rank correlation coefficients of varied parameters and HIV prevalence over time. This illustrates the relationship of each of the varied parameters with the main outcome of interest, HIV prevalence in the sexually-active population from 1990–2002. The partial rank correlation coefficient (PRCC), calculated from all runs passing the first phase of calibration, is plotted for each year between1990 and 2002. (LR: Low-risk; HR- High-risk; CSW- Commercial Sex Worker).

Despite substantial noise in the PRCC values prior to 1994, the CSW acquisition rate and the CSW multiplier showed a positive correlation with HIV prevalence at the beginning of the epidemic (Figure 4). After 1994, parameters that influence short-term (regular) partnerships (regular partnership acquisition rate, high-risk multiplier, number of acts per regular partnership per month, and proportion of males in the high-risk group) correlated strongly and positively with HIV prevalence. The pairwise heat plots (Figure S4) show that well-fitting values for each of these 4 parameters are negatively correlated with well-fitting values for the remaining influential parameters, reflecting their importance in determining prevalence and therefore fit to UNAIDS data. The PRCC values calculated for the proportion of the population with greater than 2 partnerships in the last month of 1990 (an estimate of concurrency) showed that having multiple partners positively influences the HIV prevalence for the years after 1994 (Figure S5). We also plotted the weighted average HIV prevalence curves over time stratified by age and gender (Figure S6) to confirm that our model is able to capture the difference in prevalence expected in males and females of different ages.

## Discussion

Understanding the dynamics of HIV transmission in South Africa is critical to determining the potential effect of interventions on the epidemic. This report provides a detailed description of the CEPAC Dynamic Model, which generates HIV prevalence trends over time in South Africa, based on a detailed model of the natural history of HIV infection (CEPAC). The CDM accounts for changing transmissibility over the course of HIV infection. It also incorporates sexual mixing that, while necessarily simplified from reality, incorporates known features of sexual behavior, such as heterogeneous individuals who differ in their propensity to form different types of partnerships with different durations, stochastic variation across sexual acts, the use of condoms, and possible assortativeness in partner choice. The availability of a detailed HIV infection natural history model for each infected individual, together with these forms of heterogeneity, necessitated the development of a stochastic, agent-based model. Models that include such details are parameter-rich, and the parameter values often are not directly measured in epidemiological and behavioral studies. To address these challenges, we used a Bayesian-melding-like procedure to allow for a principled way for combining prior information on demographic and behavioral parameters - both elementary quantities (e.g. rates of partnership formation) in the model, and restrictions on the composites of these quantities (e.g. prevalence of various partnership types at equilibrium before the appearance of HIV) - with HIV prevalence data in South Africa from 1990 to 2002 [1], [70].

This calibration approach incorporates prior distributions on model inputs, and estimates posterior distributions based on the fit of model outputs to prevalence data (Phase 2 calibration, Figure 3) and has many similarities with the Bayesian melding methodology described by others [25]–[28], [69], in that it also incorporated restrictions on certain behavioral properties of the population that were composite functions of the input parameters (Phase 1 calibration, Table S4), and on stratum-specific incidence and prevalence at the end of the model run, year 2002 (Phase 3 calibration, Table S5). We also noted in preliminary analyses that due to seeding of the epidemic with varying numbers of HIV-infected individuals, there was considerable stochastic variation in when the epidemic “took off”, even for a fixed parameter set, but once the epidemic exceeded a certain prevalence, repeated runs with the same parameter set gave very similar prevalence curves. Similar phenomena have been observed previously in stochastic transmission models [75] and are essentially a reflection of the law of large numbers. For this reason, we did not specify a time in the model corresponding to a particular year a priori. Rather, in the Phase 2 calibration step, we “slid” the model outputs along the UNAIDS prevalence curve to identify the model year corresponding to 1990 that gave the best fit of the data to the entire prevalence curve. Because the UNAIDS estimates contain only high and low estimates for annual prevalence, we were forced to make assumptions to translate prevalence values in the model into pseudo-likelihood weights. Despite these elaborations, each step of the approach we took was guided by the concepts of Bayesian melding.

With a complex, highly parameterized model of this sort, it is possible that very good fits to the data can be obtained with a variety of different sets of parameter values. Moreover, two parameter sets giving indistinguishable fits to the data may imply quite different trajectories for the epidemic in years after the period used to fit the model, and may imply very different impacts of proposed interventions, such as treatment or prevention interventions. Indeed, in our model, the top fitting parameter sets produce long-term dynamics that are variable in their trajectory after 2002, in the counterfactual situation of no antiretroviral therapy introduction (Figure 3). This suggests that the effort involved in calibration of a complex model should pay off in the ability to consider alternative hypotheses (i.e. alternative parameter sets) for how the epidemic reached its state in 2002, which may have different implications for the effectiveness and cost-effectiveness of different interventions post-2002. In future work, we will use the weights from these simulations to provide weighted estimates of intervention effects, reflecting these alternative possibilities for the behavioral and demographic parameters underlying the observed epidemic.

In this calibration study, we were able to assess the effects of particular parameters on the dynamics of the epidemic in a time-specific way [76]. The HIV prevalence prior to 1994 is most highly correlated with dynamics related to CSW partnerships. As the epidemic matures, the impact of CSW behavior becomes less important to the increase in prevalence than the parameters that influence short-term partnership dynamics. In the later part of the epidemic, regular partnership acquisition for low-risk males, the high-risk multiplier, the number of sexual acts per regular partnership per month, and the proportion of males who are in the high-risk group all have a positive association with HIV prevalence. Since these parameters influence the levels of number of individuals having more than one partnership in a single month in the CDM, it appears that multiple partnerships in a month, which is a proxy for concurrency, may be important to the transmission and spread of HIV in South Africa. As noted, the PRCC between the parameters and prevalence also indicate that a small core group of high-risk females and CSW is necessary to create early epidemic growth in our model. The smaller the group of high-risk individuals or CSWs, the higher the prevalence, indicating concurrency is essential to increasing prevalence in our model dynamics. This can also be seen in the PRCC relating more than one partnership in a single month and HIV prevalence over time (Figure S5).

This newly developed CDM includes extensive detail on sexual behavior, HIV infectivity, and HIV disease progression. A number of mathematical models have been developed that incorporate the potential effects of HIV prevention interventions in resource-limited settings [15]–[17], [63], [77]–[85]. While each of these models has its own strengths, the CDM builds on their foundation by simultaneously including important biological and behavioral characteristics of HIV transmission. The CDM's link to the Disease model provides a unique opportunity to provide detailed and extensive cost-effectiveness estimates for a number of interventions. Additionally, the test-and-treat pathway in the Disease Model would allow for a thorough examination of the HIV testing cascade and understanding the effects of behavior on this cascade for future analyses [86].

There are several limitations to the model. The CDM does not include the transmission of other sexually transmitted infections and their potential impact on HIV transmission. This may cause the CDM to underestimate the transmission of HIV in a population. However, the per-act transmission rates in the model were derived from cohorts that include patients co-infected with other sexually transmitted infections. In the CDM, CSWs only form CSW partnerships and therefore cannot have steady, regular or casual partnerships. Also, the CDM does not account for in-and-out-migration, which would allow for HIV-infected individuals to enter or exit the population. Migration could also allow heterogeneous mixing beyond the risk group stratification. In addition, the behavioral inputs are not varied over time. It is likely that over the thirteen-year horizon of the CDM model simulation there have been changes in sexual behaviors in South Africa. Finally, the CDM does not include mother-to-child transmission, so new births are assumed to be HIV-uninfected. Given the time frame covered by this current calibration (1990–2002), the limited access to ART among perinatally infected children in South Africa [87], and their short survival, perinatally-infected children will have a limited impact on the projections. However, as access to ART increases among these children, both their survival [88], [89] and sexual initiation [90] will need to be incorporated into the CDM.

Further, the analysis of complex individual-based simulation models is difficult within reasonable computing constraints and memory restrictions. For example, we are not able to guarantee that we spanned the entire parameter space in our random generation of parameter sets. We attempted to minimize the possibility of important dynamics outside of the parameter ranges used through extensive sensitivity analyses and mock calibrations. Many of our methods of summarizing relevant output are also limited. For example, the correlation plots represent the correlation between only two parameters at one time and not all 12. Therefore, while the noted correlations between two parameters have been observed, there may be other correlations that depend on 3- or more-way combinations of parameters that are not readily visualized in these plots. Finally, we limited the number of outputs collected from the model due to memory and computational run-time constraints.

Despite these limitations, the CDM incorporates many of the details found in previously published models into a single model for use in cost-effectiveness analyses [63], [84]. This model will be extended into the post-ART years of the HIV epidemic in South Africa; with this capability, the effect of both behavioral and treatment interventions can be assessed. Combined with rigorous literature and other data searches and a multi-step calibration procedure that incorporates information on both the behavior and biology of HIV transmission, the CDM stands as one of the most detailed models in the HIV transmission literature.

## Supporting Information

Text S1 **Programming Details and Computational Performance.**
(DOCX)

Text S2 **Probability of Transmission.**
(DOCX)

Text S3 **CDM Input Parameter Values.**
(DOCX)

Text S4 **Calibration Steps.**
(DOCX)

Text S5 **Partial Rank Correlation Coefficient (PRCC) Analysis.**
(DOCX)

Text S6 **Posterior Distributions of Model Input Parameter Values.**
(DOCX)

Table S1 **Model Input Point Estimates.**
(DOCX)

Table S2 **Summary of Disease Model input parameters.**
(DOCX)

Table S3 **Parameters for HIV Prevalence Calibration.**
(DOCX)

Table S4 **Parameters for Partnership Calibration.**
(DOCX)

Table S5 **Summary of the Phase 3 Calibration bounds.**
(DOCX)

Figure S1 **Cascade of parameter sets through the three stages of calibration.** Beginning with 264,225 randomly chosen parameter sets, each parameter set was run through the model and exposed to three sets of restrictions to determine the runs that were most realistic in terms of sexual behavior and HIV prevalence predictions. A total of 3,750 runs passed all calibration restrictions and will be used in all future model analyses.
(PDF)

Figure S2 **Cumulative runs vs. cumulative weight for runs passing the calibration procedure.** This histogram depicts the cumulative distributions of weights (for a variety of sigma values) assigned to the 3,750 runs that passed the calibration procedure. The curve with sigma = 1 corresponds to the analyses described in the main text. The cut off (sigma = 1) for the 564 runs contributing to the top 90% of the weight is denoted by a red line. As noted from the figure, as sigma increases the number of runs that contribute to the top 90% of the weight to fit increases and the variance in the weights increases as well.
(PDF)

Figure S3 **Posterior distributions for each parameter varied in the calibration procedure.** Each histogram (A–L) represents the posterior distribution for each of the parameters varied in the calibration procedure with each of the bars representing the summed weight of the runs in that parameter value bin. The panels are as follows: A. Assortativity (Assort); B. Number of sexual acts per regular partnership per month (RegActs); C. Chance of a female becoming a CSW (ChanceCSW); D. Proportion of males in HR group (PropHRMale); E. Proportion of non-CSW females in HR group (PropHRFemale); F. Partnership acquisition rate multiplier (EpsilonLR); G. HR multiplier (HRMult); H. CSW multiplier (CSWMult); I. Monthly acquisition rate for steady partnerships for LR males (AqRateStdyLR); J. Monthly acquisition rate for regular partnerships for LR males (AqRateRegLR); K. Monthly acquisition rate for casual partnerships for LR males (AqRateCasLR); and L. Monthly acquisition rate for CSW partnerships for LR males (AqRateCSWLR).
(PDF)

Figure S4 **Two-dimensional plots of model fit.** On each plot two CDM input parameters are varied, one on the x-axis and the other on the y-axis. A point was generated for each combination of the two parameter values that were part of one of the 3,750 parameter sets created in the fitting procedures. The color of each of these points represents the weight contribution for the plotted CDM model simulation with red representing parameter sets that contribute the top 90% of the weight and cyan representing parameter sets that contribute the bottom 10% of the weight. The plots represent the correlation between every parameter varied in the fitting procedures. The figure abbreviations are as follows: high-risk multiplier (HRMult), the CSW-risk multiplier (CSWmult), assortativeness parameter (Assort), proportion of males in the high-risk group (PropHRMale), proportion of females in the high-risk group (PropHRFemale), partner acquisition rate multiplier while in steady partnership (EpsilonLR), monthly partner acquisition rate for a steady partnership among low-risk males (AqRateStdyLR), monthly partner acquisition rate for a regular partnership among low-risk males (AqRateRegLR), monthly partner acquisition rate for a casual partnership among low-risk males (AqRateCasLR), monthly partner acquisition rate for a CSW encounter among low-risk males (AqRateCSWLR), chance of a female becoming a CSW (P(CSW_F_)), and number of acts per regular partnership per month among low and high-risk males (RegActs). The panels are as follows: A. The correlation between the monthly partner acquisition rate for a regular partnership among low-risk males (AqRateRegLR) and the 12 varied parameters; B. The correlation between the high-risk multiplier (HRMult) and the 12 varied parameters; C. The correlation between the proportion of males in the high-risk group (PropHRMale) and the 12 varied parameters; and D, The correlation between the number of acts per regular partnership per month among low and high-risk males (RegActs) and the 12 varied parameters. We have added random jitter around each discrete value for RegActs so that one can see points on the plot.
(PDF)

Figure S5 **Partial rank correlation coefficients of concurrency in 1990 and HIV prevalence over time.** This graph illustrates the relationship between the proportion of the population with greater than 2 partnerships in the last month of 1990 and the main outcome of interest, HIV prevalence in the sexually active population from 1990–2002. The partial rank correlation coefficient (PRCC) is plotted for each year between 1990–2002. The PRCC summarizes the impact of concurrency on the HIV prevalence in that year. A positive PRCC indicates that the HIV prevalence is positively correlated with concurrency (and inversely so for a negative PRCC). Despite a great deal of noise in the years prior to 1994, concurrency (defined as having 2 or more partners in the last month of 1990) positively influences the HIV prevalence for years after 1994.
(TIFF)

Figure S6 **The CDM predicted HIV prevalence in South Africa from 1990–2002 stratified by age and gender.** These graphs depict the weighted average of the HIV epidemic curves generated by the CDM, from the parameter sets that contribute the top 90% of the likelihood weight, stratified by gender and age. The panels are as follows: A. Depicts the weighted average HIV prevalence for males and females; B. Depicts the weighted average HIV prevalence for males stratified by the 8 age buckets modeled in the CDM; C. Depicts the weighted average HIV prevalence for females stratified by the 8 age buckets modeled in the CDM.
(PDF)
