# Correction: Development, Calibration and Performance of an HIV Transmission Model Incorporating Natural History and Behavioral Patterns: Application in South Africa

**DOI:** 10.1371/journal.pone.0106212

**Published:** 2014-08-19

**Authors:** The PLOS ONE Staff

In the Funding section, the second grant number is incorrect. The correct Funding statement is: This work was funded by the following grants: RO1-AI-058736, RO1-MH-087328, T32-AI-007535 and T32-AI-007433. The funders had no role in study design, data collection and analysis, decision to publish, or preparation of the manuscript.
