# Supplementary material for: Development, Calibration and Performance of an HIV Transmission Model Incorporating Natural History and Behavioral Patterns: Application in South Africa
Source: PLoS One. 2014 May 27;9(5):e98272. doi: 10.1371/journal.pone.0098272 (PMC4035281; doi:10.1371/journal.pone.0098272)
Supplement: Text S1 — Programming Details and Computational Performance. (DOCX) [file pone.0098272.s001.docx]

**Text S1: Programming Details and Computational Performance**

The CDM is written entirely in C++ using modern paradigms and design patterns. The code is cross-platform compatible working equivalently on Windows (XP, 7, and 8.1), OSX (10.9) and Linux for 32- and 64-bit architectures. It is compiled using the primary compiler for each platform: MSVC 12, Clang 5.0.2, and GCC 4.8.2 respectively with full optimization.

With a population of size *n* for every time step, each male can form a partnership with a random number of females in the population depending on his partnership acquisition rate. As no one can have multiple partnerships with the same person within a month and partnerships last at least one time step, the number of partnerships formed per unit of time is worst-case *n(n+1)/2 (O(n^2^))*. The partnership formation algorithm itself evaluates partners based on a set of selection criteria. In the worst case, every individual in the population may be evaluated for those criteria before an individual partnership is formed or not formed. Thus, the entire partnership formation step is *n(n(n+1)/2) (O(n^3^))*. All other updates in a time step are *O(n)* or *O(n^2^)* as a function of population size so the time complexity of a single time step is bounded by *O(n^3^)*.

In our simulation, population increases exponentially (here 3% annually). At some point in the simulation, generally at the end, population size will reach a maximum, *n_max_*. By definition, at all other time steps, the population size will be smaller than this maximum. Overall running time is therefore O(tn_max_^3^). As long as one individual can form a partnership with some unbounded subset of the entire population, this time complexity is necessarily optimal.

For a single simulation run, individual time steps could be divided across multiple processors in parallel thereby reducing computation time by a constant factor, n_proc_. This approach was considered, however, the need to run several thousand independent simulations for Bayesian melding meant that parallelizing one simulation across multiple processors and running each simulation separately and simultaneously on those selfsame processors would take approximately the same amount of time. We therefore chose to keep each simulation single-threaded. Speedups may be realized by prematurely stopping runs that reach an unrealistic state (See Calibration Steps Text S4), reducing the simulation duration, limiting population size, or some combination thereof. We do all of these when possible.

Our starting population size is generally 100,000 and *n_max_* is usually less than 750,000. *t* is often 1200. Overall running time varies based on actual simulation parameters and hardware but tends to be on the order of a few hours (3 to 6) for one run with the first months taking less than one second of computation time and the last months taking 10 to 20 seconds each.

All parts of the model operate on a one-month time step. In general this time step is not inherent to the simulation framework but is instead determined by the parameters used (e.g. monthly partnership acquisition rate, etc.) and calibration procedure. Few data exist for high-level statistics such as yearly population prevalence and fewer still for monthly period or more specific behavioral parameters. It is assumed that, with correct inputs, a simulation of 365 days would be effectively equivalent to a simulation of twelve aggregate months, which would also be equivalent to one simulated aggregate year. One month was chosen as a useful compromise between available data, simulation time, and generalizability.
