# Supplementary material for: Development, Calibration and Performance of an HIV Transmission Model Incorporating Natural History and Behavioral Patterns: Application in South Africa
Source: PLoS One. 2014 May 27;9(5):e98272. doi: 10.1371/journal.pone.0098272 (PMC4035281; doi:10.1371/journal.pone.0098272)
Supplement: Text S2 — Probability of Transmission. (DOCX) [file pone.0098272.s002.docx]

**Text S2: Probability of Transmission**

The probability of HIV transmission at each sexual act with an HIV infected individual (PTrAct) in the CDM is dependent upon the following parameter values: the *probability of transmission per act* (β_HVL_), which is dependent on an infected individual’s HIV RNA and stage of HIV infection (see Table S1); the *per-act probability of condom use by partner type* (con_p_); the *protective efficacy of a condom* *(*protEffCon); *circumcision status* (circ_i_); and the *protective efficacy of circumcision* (protEffCirc). PTrAct increases as the HIV RNA level increases and it is decreased by circumcision and condom use. Therefore, the PTrAct for HIV-infected males transmitting to an uninfected female (MtoF) is:

PTrAct_MtoF_ = β_HVL_*[1-(con_p_*protEffCon)]

and PTrAct for HIV-infected females transmitting to an uninfected male (FtoM) is:

PTrAct_FtoM_ = β_HVL_*[1-(con_p_*protEffCon)]*(1-Circ_i_*protEffCirc).

From this, the probability of transmission per month (PTrMonth) for each partnership is calculated using the following equation:

PTrMonth = 1-(1-PTrAct)^n^

where n is the number of acts per partnership per month and PTrAct is either MtoF or FtoM depending on which partner is infected.
