# Supplementary material for: Development, Calibration and Performance of an HIV Transmission Model Incorporating Natural History and Behavioral Patterns: Application in South Africa
Source: PLoS One. 2014 May 27;9(5):e98272. doi: 10.1371/journal.pone.0098272 (PMC4035281; doi:10.1371/journal.pone.0098272)
Supplement: Text S3 — CDM Input Parameter Values. (DOCX) [file pone.0098272.s003.docx]

**Text S3. CDM Input parameter values**

Table S1 provides values for the CDM input parameters that are not varied in the calibration procedure and Table S2 provides values for the CEPAC (natural history of HIV infection) parameter values that are important for the CDM analyses; these are also fixed in our analyses.

Despite a thorough review of the literature, the initial CD4 count for incident HIV infected cases was uncertain. Initial CD4 counts ranged from 503 to 2051 cells/mm^3^ based on differing populations, study designs, and assumptions [[1-19](#_ENREF_1)]. We varied this number in our model and observed the changes in the life expectancy (time from infection to death) for HIV positive people in order to determine the best initial CD4 count estimate. In our sensitivity analyses an incident initial CD4 count of 442 cells/mm^3^ resulted in a life expectancy for HIV positive people of 4.9 years, 884 cells/mm^3^ in a life expectancy of 8.5 years, and 1200 cells/mm^3^ of 9.8 years. Based on survival estimates by Bachmann et al. [[20](#_ENREF_20)] of 8.2 years, we decided to assign the initial CD4 count for incident HIV infections to 884 cells/mm^3^.

**References:**

1. Williams, B.G., et al., *HIV infection, antiretroviral therapy, and CD4+ cell count distributions in African populations.* The Journal of Infectious Diseases, 2006. **194**(10): p. 1450-1458.

2. Lawrie, D., et al., *Local reference ranges for full blood count and CD4 lymphocyte count testing.* S Afr Med J, 2009. **99**(4): p. 243-8.

3. Korenromp, E.L., et al., *Clinical prognostic value of RNA viral load and CD4 cell counts during untreated HIV-1 infection--a quantitative review.* PLOS ONE, 2009. **4**(6): p. e5950.

4. Maini, M.K., et al., *Reference ranges and sources of variability of CD4 counts in HIV-seronegative women and men.* Genitourinary Medicine, 1996. **72**(1): p. 27-31.

5. Re, M.C., et al., *HIV-1 DNA proviral load in treated and untreated HIV-1 seropositive patients.* Clinical Microbiology and Infection, 2010. **16**(6): p. 640-6.

6. Mair, C., et al., *Factors associated with CD4 lymphocyte counts in HIV-negative Senegalese individuals.* Clinical and Experimental Immunology, 2008. **151**(3): p. 432-40.

7. Kassa, E., et al., *Evaluation of the World Health Organization staging system for HIV infection and disease in Ethiopia: association between clinical stages and laboratory markers.* AIDS, 1999. **13**(3): p. 381-9.

8. Kassu, A., et al., *Distribution of lymphocyte subsets in healthy human immunodeficiency virus-negative adult Ethiopians from two geographic locales.* Clinical and Diagnostic Laboratory Immunology 2001. **8**(6): p. 1171-6.

9. Lisse, I.M., et al., *Serial CD4 and CD8 T-lymphocyte counts and associated mortality in an HIV-2-infected population in Guinea-Bissau.* Journal of Acquired Immune Deficiency Syndromes, 1996. **13**(4): p. 355-62.

10. Ricard, D., et al., *The effects of HIV-2 infection in a rural area of Guinea-Bissau.* AIDS, 1994. **8**(7): p. 977-82.

11. Aina, O., et al., *Reference values of CD4 T lymphocytes in human immunodeficiency virus-negative adult Nigerians.* Clinical and Diagnostic Laboratory Immunology, 2005. **12**(4): p. 525-30.

12. Auvert, B., et al., *Can highly active antiretroviral therapy reduce the spread of HIV?: A study in a township of South Africa.* Journal of acquired immune deficiency syndromes, 2004. **36**(1): p. 613-21.

13. Levin, A., et al., *Determination of T-lymphocyte subsets on site in rural Tanzania: results in HIV-1 infected and non-infected individuals.* International Journal of STD and AIDS, 1996. **7**(4): p. 288-91.

14. Lugada, E.S., et al., *Population-based hematologic and immunologic reference values for a healthy Ugandan population.* Clinical and Diagnostic Laboratory Immunology 2004. **11**(1): p. 29-34.

15. Urassa, W., et al., *Rate of decline of absolute number and percentage of CD4 T lymphocytes among HIV-1-infected adults in Dar es Salaam, Tanzania.* AIDS, 2004. **18**(3): p. 433-8.

16. Tugume, S.B., et al., *Hematological reference ranges among healthy Ugandans.* Clinical and Diagnostic Laboratory Immunology, 1995. **2**(2): p. 233-5.

17. Kelly, P., et al., *Morbidity and nutritional impairment in relation to CD4 count in a Zambian population with high HIV prevalence.* Acta Tropica, 2002. **83**(2): p. 151-8.

18. Phair, J.P., et al., *Detection of infection with human immunodeficiency virus type 1 before seroconversion: correlation with clinical symptoms and outcome.* The Journal of Infectious Diseases, 1997. **175**(4): p. 959-62.

19. Granich, R.M., et al., *Universal voluntary HIV testing with immediate antiretroviral therapy as a strategy for elimination of HIV transmission: a mathematical model.* The Lancet, 2009. **373**(9657): p. 48-57.

20. Bachmann, M.O., *Effectiveness and cost effectiveness of early and late prevention of HIV/AIDS progression with antiretrovirals or antibiotics in Southern African adults.* AIDS Care, 2006. **18**(2): p. 109-20.
