# Supplementary material for: Development, Calibration and Performance of an HIV Transmission Model Incorporating Natural History and Behavioral Patterns: Application in South Africa
Source: PLoS One. 2014 May 27;9(5):e98272. doi: 10.1371/journal.pone.0098272 (PMC4035281; doi:10.1371/journal.pone.0098272)
Supplement: Text S4 — Calibration Steps. (DOCX) [file pone.0098272.s004.docx]

**Text S4: Calibration Steps**

Below we describe the three phases of calibration in detail. The goal of the calibration procedure was to produce model runs that replicated data on sexual behavior, partnership histories, and HIV prevalence in South Africa.

**S4.1. Phase I – Behavioral Calibration**

The goal of the first calibration step is to determine whether a parameter set produces realistic patterns of sexual partnerships and acts, based on prior knowledge and an extensive literature review of South African data. This calibration step is necessary because many model inputs that affect partnership prevalence and sexual behavior (i.e. partnership formation rates) have not been directly measured in the literature, but many model outputs (e.g. the proportion of the population in particular partnership types) have been measured and reported. Acceptable ranges (comparable to uniform priors) were specified for five behavioral outputs, and then an additional four constraints were imposed in the form of inequalities between male and female behavioral parameters. We set to zero the weights of parameter sets that produced model output that fell outside these limits, for **any one of more** of the constraints, effectively discarding these parameter sets. The parameter sets whose output fell within all constraints were considered to pass “phase 1” or “behavioral” calibration.

Phase 1 calibration restricted the model output using pre-specified prior distributions (defined in Table S4), of the following quantities:

- The proportion of the **entire sexually active (SA) population** that was in a steady partnership (non-single) the year prior to Phase 1 calibration (the last year of model initialization)
- The proportion of **SA males** that had at least one casual partnership the year prior to Phase 1 calibration (the last year of model initialization)
- The proportion of **SA males** that had at least one CSW partnership the year prior to Phase 1 calibration (the last year of model initialization)
- The proportion of **SA** **males** with more than 1 partner (one of which was a steady/regular partnership) the year prior to Phase 1 calibration (the last year of model initialization)
- The average number of acts per person among the **entire SA population** in the last month of model initialization

In addition, there were conditions specific to sexually active females only:

- The casual partnership prevalence among sexually active females could be **no greater than** that of sexually active males.
- The percent of sexually active females in multiple partnerships within the past month could be **no greater than** that of sexually active males.
- The ratio of acts per month for low risk sexually active females could be **no greater than** that of high risk females, multiplied by some user-defined ratio.

For the analyses reported here, these user-defined ratios were all set to one.

Phase 1 calibration examined model output after the model initialization period (currently set to 50 years). If the partnership outcomes did not satisfy all of the above-mentioned criteria, the parameter set was assign zero weight and HIV was not introduced for that parameter set. The ranges used for the prior output restrictions are shown in Table S4.

**S4.2 Phase 2 - HIV Prevalence Calibration**

Once all parameter sets that satisfied the behavioral data restrictions (Phase 1) were identified and HIV was introduced to each individual run, it was important to determine how well the epidemic curve produced by the CDM for each parameter set fit the UNAIDS data (Phase 2). After we produced a HIV prevalence curve for a parameter set, the model identified the section of the HIV prevalence curve that best fit the UNAIDS HIV prevalence data from 1990-2002 (Table S3), by “sliding” the HIV output prevalence along the UNAIDS data (on the x-axis) until the difference between the 13 time points was as small as possible. This is equivalent to identifying which 13 year period of the model output corresponded to the best fit to the UNAIDS data from 1990-2002.

To do the fit, we used the Levenberg-Marquardt algorithm (LMA), which is a standard iterative fitting procedure that seeks to minimize the value of the sum of squares, i.e. , where $y_{i}$ was the UNAIDS HIV prevalence for year *i, M* was the HIV prevalence from the model run, *t* was the number of months the model had run since the end of model initialization and *a* was the fit parameter representing the number of months we shifted the model run to the UNAIDS HIV prevalence data points. From the fitting procedure we extracted two pieces of information: a list of the rankings of the best fitting runs based on the LMA weights and for each run, the model time points corresponding the period that provided the best fit to the 1990-2002 UNAIDS data.

**S4.3. Phase 3- Reality Checks and Likelihood Weighting**

Once the prevalence output for each parameter set had been calibrated to UNAIDS data, further filtering was performed to assess whether the behavioral patterns at the end of the calibration period (2002) were as expected based on the literature. We analyzed the parameter set runs that had passed Phase 1 and Phase 2 calibration and determined if the outputs for each run fell within the ranges gathered from the literature for the quantities outlined in Table S5. Any parameter set producing a value for any quantity that fell outside these ranges had its weight set to zero and was not considered further.

For the parameter sets that passed the preceding steps (thus had not had zero weights assigned), pseudo-likelihood weights were produced for each to indicate how well it fit the UNAIDS HIV prevalence data being used for calibration [[1](#_ENREF_1)]. Since we ran the model for 50 years with HIV and needed at least 12 years for the curve to fit the years from 1990 to 2002, runs that took longer than 38 years to produce an epidemic were assigned zero weight; although their fit would have led to very low weights in any case, we chose to set them to zero to reduce computational time in future work.

The likelihood for run *j* that passed Phases 1, 2 and 3 of calibration was given by:

where *X_ij_* is the HIV prevalence of year *i* from run *j*, is the high estimate for the UNAIDS HIV prevalence for year i, is the low end estimate for the UNAIDS HIV prevalence for year i, Y_i_ is the UNAIDS HIV prevalence of year i, and σ is an adjustment factor that accounts for the uncertainty in the UNAIDS high and low estimates. We initially assumed σ = 1 but were able to adjust this according to observed output, as explained below.

We normalized the weights such that for run j:

We compared the calculated likelihood weights for each run against the run’s fit to evaluate whether factor adjustments were necessary. As expected, when the adjustment factor was increased, we observed a wider spread in the likelihood weights, allowing more runs to contribute to the fit of the HIV prevalence curve. After examining the effects of varying the adjustment factor, σ, across a wide range of values (0-2.5), we decided that σ=1 was the best choice since with this value the runs that contributed 90% of the weight approximated the UNAIDS curve fell almost entirely within the upper and lower UNAIDS estimates. Of the 3,750 runs that passed the calibration procedure with nonzero weights, the best-fitting 564 runs contributed 90% of the weight (Figure S2).

**References:**

1. UNAIDS. *AIDSinfo Online Database - South Africa*. 2012 [cited 2013 August 1]; Available from: <http://www.unaids.org/en/dataanalysis/datatools/aidsinfo/>.
