# Supplementary material for: Development, Calibration and Performance of an HIV Transmission Model Incorporating Natural History and Behavioral Patterns: Application in South Africa
Source: PLoS One. 2014 May 27;9(5):e98272. doi: 10.1371/journal.pone.0098272 (PMC4035281; doi:10.1371/journal.pone.0098272)
Supplement: Text S5 — Partial Rank Correlation Coefficient (PRCC) Analysis. (DOCX) [file pone.0098272.s005.docx]

**Text S5. Partial Rank Correlation Coefficient (PRCC) Analysis**

To examine the influence of the CDM parameters on HIV prevalence, partial rank correlation coefficients (PRCC) were calculated between each of the 12 parameters that were varied in the calibration procedures (as well as a composite measure of the concurrency at the beginning of each year, in a separate analysis) and the HIV prevalence every year from 1990-2002 (Table 1) [[1](#_ENREF_1)]. In our analyses we have defined concurrency as having two or more partners of any type within a month. The PRCC were calculated to understand the impact of each parameter (controlling for all others) on the prevalence over time. A positive PRCC describes a positive correlation between the value of the parameter in a given run and the prevalence at a particular year (and inversely so for negative PRCC). The concurrency PRCC was calculated to understand the impact of multiple partnerships in a single month on the HIV prevalence over time. We would expect, and observed, that parameters that positively correlated to HIV prevalence were related to concurrency, and subsequently, that concurrency measured directly, is related to HIV prevalence. As can be seen from Figure 4 and Figure S5, it appears to be the case that concurrency does indeed contribute to the HIV prevalence at each time point.

**References:**

1. Blower S, Dowlatabadi H (1994) Sensitivity and uncertainty analysis of complex models of disease transmission: an HIV model, as an example. International Statistical Review 62: 229-243.
