# Supplementary material for: Development, Calibration and Performance of an HIV Transmission Model Incorporating Natural History and Behavioral Patterns: Application in South Africa
Source: PLoS One. 2014 May 27;9(5):e98272. doi: 10.1371/journal.pone.0098272 (PMC4035281; doi:10.1371/journal.pone.0098272)
Supplement: Text S6 — Posterior Distributions of Model Input Parameter Values. (DOCX) [file pone.0098272.s006.docx]

**Text S6: Posterior distributions of model input parameter values**

Estimated marginal posterior distributions of the model input parameters are shown in Figure S3, which are simply histograms of the parameter values, weighted by the total likelihood weight of values within each bin of the histogram. High values of this ratio correspond to parameters strongly constrained (marginally) by the data, most notably the four types of partnership acquisition rates for low risk males, the proportion of high-risk males, and the high-risk multiplier that determines the increased sexual activity of high-risk males. This is unsurprising, since these were the parameters that were most strongly correlated with the prevalence used for fitting (Figure 4).

To visualize these posterior distributions for pairs of parameters, we created heat plots for each pair of parameters, in which the runs contributing the top 90% of weight are shown in red, the remaining nonzero-weighted runs are shown in cyan, and runs not passing one step of calibration (zero weight) are not shown. For comparison, the prior distribution of these parameters was multivariate normal, so any pattern visualized in these plots relative to a random distribution across the plane shows an effect of the calibration in favoring certain parameter values over others.

We have included four sets of correlation plots that have the most apparent two-way patterns (regular acquisition rate, high risk multiplier, regular acts, and the proportion of men who are high risk) (Figure S4). In addition to those discussed in the results section, there were several additional patterns visible. Parameter sets with high or low values for both the acquisition rate of CSW partners by low-risk men, and the proportion of high risk men, were eliminated by the calibration steps because they produced either too few, or too many acts with CSWs (visible as white space at the upper right and lower left of the corresponding plot). A similar pattern is apparent for the pair the acquisition rate of regular partners for low-risk men and the high-risk multiplier, presumably for similar reasons, and here, moreover, even among the sets that passed behavioral calibration, the best fitting runs fell within a narrower range prescribing a higher value for one parameter if the other was low. There is a slight negative association between the CSW-risk multiplier and the CSW acquisition rate, suggesting that when the CSW acquisition rate for low risk men is small, the CSW-risk multiplier needs to be larger to fuel the epidemic among high-risk men. While there are no strong relationships that are inexplicable in these plots, some expected correlations (for example, a tendency for CSW acquisition rates to trade off with the high-risk multiplier) were not observed. Just as two-dimensional plots provide insights less apparent on marginal univariate posterior histograms, more subtle relationships might become visible if it were possible to visualize relationships among three or more parameter values.
