# Supplementary material for: Development, Calibration and Performance of an HIV Transmission Model Incorporating Natural History and Behavioral Patterns: Application in South Africa
Source: PLoS One. 2014 May 27;9(5):e98272. doi: 10.1371/journal.pone.0098272 (PMC4035281; doi:10.1371/journal.pone.0098272)
Supplement: Table S1 — Model Input Point Estimates. (DOCX) [file pone.0098272.s007.docx]

**Table S1: Model Input Point Estimates**

| **Parameter Description** | **Point Estimate** | | | | **Source** |
| --- | --- | --- | --- | --- | --- |
| Initial number of prevalent HIV cases | 6 | | | | MA |
| Distribution of initial HIV prevalent cases | 1 for HR males aged 17-19 years | | | | MA |
|  | 1 for HR males aged 20-24 | | | |  |
|  | 1 for HR females aged 17-19 | | | |  |
|  | 1 for HR females aged 20-24 | | | |  |
|  | 1 for female CSW aged 17-19 | | | |  |
|  | 1 for female CSW aged 20-24 | | | |  |
|  |  | | | |  |
| **Population Characteristics** |  | | | |  |
| Population size at model initiation | 100,000 | | | | User Defined |
| Number of births per person per month | 0.0022 | | | | [[1](#_ENREF_1)]* |
| Proportion of the population who is male | 0.50 | | | | MA, [[1](#_ENREF_1)] |
| Age distribution for males (proportion, age range in years) | 0.43, 0-16 | | | | [[1](#_ENREF_1)] |
|  | 0.062, 17-19 | | | |  |
|  | 0.091, 20-24 | | | |  |
|  | 0.082, 25-29 | | | |  |
|  | 0.073, 30-34 | | | |  |
|  | 0.061, 35-39 | | | |  |
|  | 0.050, 40-44 | | | |  |
|  | 0.040, 45-49 | | | |  |
|  | 0.11, 50+ | | | |  |
| Age distribution for females (proportion, age range in years) | 0.41, 0-16 | | | | [[1](#_ENREF_1)] |
|  | 0.059, 17-19 | | | |  |
|  | 0.089, 20-24 | | | |  |
|  | 0.082, 25-29 | | | |  |
|  | 0.074, 30-34 | | | |  |
|  | 0.063, 35-39 | | | |  |
|  | 0.052, 40-44 | | | |  |
|  | 0.042, 45-49 | | | |  |
|  | 0.13, 50+ | | | |  |
| Proportion of the male population who are circumcised | 0.35 | | | | [[2-5](#_ENREF_2)] |
| Age of first sex | 17 years | | | | [[2](#_ENREF_2),[4](#_ENREF_4),[6](#_ENREF_6)] |
| Chance of a male becoming a CSW | 0 | | | | MA |
| Age range for being CSW | 17-70 years old | | | | MA |
| Proportion of CSW females in the HR group | 1 | | | | MA |
|  |  | | | |  |
| **Sexual Partnership Characteristics** |  | | | |  |
| *Steady Partnerships* |  | | | |  |
| Duration of partnership (for HR and LR males) | 10.20 years (SD 7.8 years) | | | | [[7-9](#_ENREF_7)] |
| Number of sexual acts per partnership per month (for HR and LR) | 9 | | | | [[10-12](#_ENREF_10)] |
| Probability of male condom use per month (for HR and LR males) | 0.12 (SD 0.06) | | | | [[6](#_ENREF_6),[13](#_ENREF_13),[14](#_ENREF_14)] |
| *Regular Partnership* |  | | | |  |
| Duration of partnership (for high and low risk males) | 13.50 months (SD 9 months) | | | | [[15](#_ENREF_15),[16](#_ENREF_16)] |
| Probability of male condom use per month (for HR and LR males) | 0.29 (SD 0.15) | | | | MA, [[6](#_ENREF_6),[16-18](#_ENREF_16)] |
| *Casual Partnership* |  | | | |  |
| Duration of casual partnership (for HR and LR males) | 0 | | | | MA |
| Number of acts per partnership per month (for HR and LR males) | 1 | | | | MA |
| Probability of male condom use (for HR and LR males) | 0.37 (SD 0.19) | | | | MA, [[6](#_ENREF_6),[16](#_ENREF_16),[17](#_ENREF_17)] |
| *CSW Encounters* |  | | | |  |
| Duration of CSW encounter | 0 | | | | MA |
| Number of acts per partnership per month (for HR and LR males) | 1 | | | | MA |
| Probability of male condom use (for HR and LR males) | 0.50 (SD 0.25) | | | | MA, [[13](#_ENREF_13)] |
|  |  | | | |  |
| **Partner Acquisition rate modifiers** |  | | | |  |
| Starting age of discounting | 50 | | | | MA |
| Age discounting factor for partnership acquisition per year | 0.10 | | | | MA, [[19](#_ENREF_19),[20](#_ENREF_20)] |
| Age discounting factor for coital acts per year | 0.10 | | | | MA, [[19](#_ENREF_19),[20](#_ENREF_20)] |
|  |  | | | |  |
| **Partnership Selection Criteria** |  | | | |  |
| Average number of years younger the female is compared to male partner (steady, regular and casual) | 5 (SD 2.50) | | | | [[7](#_ENREF_7),[14](#_ENREF_14)] |
| Average number of years younger CSW is compared to male partner | 13 (SD 6.50) | | | | [[21-23](#_ENREF_21)] |
|  |  | | | |  |
| **Partner Selection Weights** |  | | | |  |
|  | Type of partnership being formed | | | |  |
| Type of female to be selected | Steady | Regular | Casual | CSW | MA |
| Single | 1 | 0.95 | 0.95 | 0 |  |
| Non-Single | 0 | 0.05 | 0.05 | 0 |  |
| CSW | 0 | 0 | 0 | 1 |  |
|  |  | | | |  |
| **Probability of Transmission** |  | | | |  |
| HIV RNA (copies/ml) | Probability of transmission per sexual act (β_HVL_) | | | | [[11](#_ENREF_11),[12](#_ENREF_12),[24](#_ENREF_24),[25](#_ENREF_25)] |
| 0-500 | 0.00010 | | | |  |
| 501-3,000 | 0.0012 | | | |  |
| 3,001-10,000 | 0.0012 | | | |  |
| 10,001-30,000 | 0.0014 | | | |  |
| 30,001 + | 0.0023 | | | |  |
| Primary infection | 0.0082 | | | | [[12](#_ENREF_12)] |
| Late-stage infection | 0.0036 | | | | [[12](#_ENREF_12)] |
|  |  | | | |  |
| Protective Efficacy of Circumcision | 0.56 | | | | [[26-29](#_ENREF_26)] |
| Protective Efficacy of Condoms | 0.80 | | | | [[30](#_ENREF_30)] |
|  |  | | | |  |

*****Calculated as an average of the birth rates from 1985-2002

**Table abbreviations:** MA=model assumption, CSW= commercial sex worker, and HR= high risk, LR=low risk

**References:**

1. U.S Census Bureau (2012) International Data Base Demographic Overview for South Africa.

2. Pettifor AE, Rees HV, Kleinschmidt I, Steffenson AE, MacPhail C, et al. (2005) Young people's sexual health in South Africa: HIV prevalence and sexual behaviors from a nationally representative household survey. AIDS 19: 1525-1534.

3. Connolly C, Simbayi LC, Shanmugam R, Nqeketo A (2008) Male circumcision and its relationship to HIV infection in South Africa: results of a national survey in 2002. South African Medical Journal 98: 789-794.

4. Shisana O, Simbayi LC (2002) Nelson Mandela/HSRC Study of HIV/AIDS: South African National HIV Prevalence, Behavioural Risks and Mass Media: Household Survey 2002. Cape Town: Human Sciences Research Council Publishers.

5. Rain-Taljaard RC, Lagarde E, Taljaard DJ, Campbell C, MacPhail C, et al. (2003) Potential for an intervention based on male circumcision in a South African town with high levels of HIV infection. AIDS Care 15: 315-327.

6. Department of Health (2002) South Africa Demographic and Health Survey 1998 - Full Report.

7. Lurie MN, Williams BG, Zuma K, Mkaya-Mwamburi D, Garnett GP, et al. (2003) Who infects whom? HIV-1 concordance and discordance among migrant and non-migrant couples in South Africa. AIDS 17: 2245-2252.

8. Ndase P, Celum C, Thomas K, Donnell D, Fife KH, et al. (2012) Outside sexual partnerships and risk of HIV acquisition for HIV uninfected partners in African HIV serodiscordant partnerships. Journal of Acquired Immune Deficiency Syndromes 59: 65-71.

9. Statistics South Africa (2008) Marriages and divorces 2007. Statistical Release P0307.

10. Skoler-Karpoff S, Ramjee G, Ahmed K, Altini L, Plagianos MG, et al. (2008) Efficacy of Carraguard for prevention of HIV infection in women in South Africa: a randomised, double-blind, placebo-controlled trial. The Lancet 372: 1977-1987.

11. Gray RH, Wawer MJ, Brookmeyer R, Sewankambo NK, Serwadda D, et al. (2001) Probability of HIV-1 transmission per coital act in monogamous, heterosexual, HIV-1-discordant couples in Rakai, Uganda. The Lancet 357: 1149-1153.

12. Wawer MJ, Gray RH, Sewankambo NK, Serwadda D, Li X, et al. (2005) Rates of HIV-1 transmission per coital act, by stage of HIV-1 infection, in Rakai, Uganda. The Journal of Infectious Diseases 191: 1403-1409.

13. Meekers D (2000) Going underground and going after women: trends in sexual risk behaviour among gold miners in South Africa. International Journal of STD and AIDS 11: 21-26.

14. Maharaj P, Cleland J (2005) Risk perception and condom use among married or cohabiting couples in KwaZulu-Natal, South Africa. International Family Planning Perspectives 31: 24-29.

15. Jewkes R, Vundule C, Maforah F, Jordaan E (2001) Relationship dynamics and teenage pregnancy in South Africa. Social Science and Medicine 52: 733-744.

16. Pettifor AE (2005) Young people's sexual health in South Africa: HIV prevalence and sexual behaviors from a nationally representative household survey. Personally distributed dataset.

17. Hargreaves JR, Bonell CP, Morison LA, Kim JC, Phetla G, et al. (2007) Explaining continued high HIV prevalence in South Africa: socioeconomic factors, HIV incidence and sexual behaviour change among a rural cohort, 2001-2004. AIDS 21: S39-48.

18. Magnani R, Macintyre K, Karim AM, Brown L, Hutchinson P, et al. (2005) The impact of life skills education on adolescent sexual risk behaviors in KwaZulu-Natal, South Africa. Journal of Adolescent Health 36: 289-304.

19. Lindau ST, Schumm LP, Laumann EO, Levinson W, O'Muircheartaigh CA, et al. (2007) A study of sexuality and health among older adults in the United States. The New England Journal of Medicine 357: 762-774.

20. Peltzer K, Phaswana-Mafuya N, Mzolo T, Tabane C, Zuma K (2010) Sexual behaviour, HIV status and HIV risk among older South Africans. Journal of Ethnobiology and Ethnomedicine 4: 163-172.

21. Allen S, Meinzen-Derr J, Kautzman M, Zulu I, Trask S, et al. (2003) Sexual behavior of HIV discordant couples after HIV counseling and testing. AIDS 17: 733-740.

22. Karim QA, Karim SS, Soldan K, Zondi M (1995) Reducing the risk of HIV infection among South African sex workers: socioeconomic and gender barriers. American Journal of Public Health 85: 1521-1525.

23. Ramjee G, Gouws E (2002) Prevalence of HIV among truck drivers visiting sex workers in KwaZulu-Natal, South Africa. Sexually Transmitted Diseases 29: 44-49.

24. Boily MC, Baggaley RF, Wang L, Masse B, White RG, et al. (2009) Heterosexual risk of HIV-1 infection per sexual act: systematic review and meta-analysis of observational studies. The Lancet Infectious Diseases 9: 118-129.

25. Powers KA, Poole C, Pettifor AE, Cohen MS (2008) Rethinking the heterosexual infectivity of HIV-1: a systematic review and meta-analysis. The Lancet Infectious Diseases 8: 553-563.

26. Mills E, Cooper C, Anema A, Guyatt G (2008) Male circumcision for the prevention of heterosexually acquired HIV infection: a meta-analysis of randomized trials involving 11,050 men. HIV Medicine 9: 332-335.

27. Auvert B, Taljaard D, Lagarde E, Sobngwi-Tambekou J, Sitta R, et al. (2005) Randomized, controlled intervention trial of male circumcision for reduction of HIV infection risk: the ANRS 1265 Trial. PLOS Medicine 2: e298.

28. Gray RH, Kigozi G, Serwadda D, Makumbi F, Watya S, et al. (2007) Male circumcision for HIV prevention in men in Rakai, Uganda: a randomised trial. The Lancet 369: 657-666.

29. Bailey RC, Moses S, Parker CB, Agot K, Maclean I, et al. (2007) Male circumcision for HIV prevention in young men in Kisumu, Kenya: a randomised controlled trial. The Lancet 369: 643-656.

30. Weller S, Davis K (2002) Condom effectiveness in reducing heterosexual HIV transmission. The Cochrane database of systematic reviews: CD003255.
