# Supplementary material for: Development, Calibration and Performance of an HIV Transmission Model Incorporating Natural History and Behavioral Patterns: Application in South Africa
Source: PLoS One. 2014 May 27;9(5):e98272. doi: 10.1371/journal.pone.0098272 (PMC4035281; doi:10.1371/journal.pone.0098272)
Supplement: Table S2 — Summary of Disease Model input parameters. (DOCX) [file pone.0098272.s008.docx]

**Table S2: Summary of Disease Model input parameters.**

| **Mean initial values for CD4 cell count ^1^** |  |  |
| --- | --- | --- |
| Acute | 884 cells/mm^3^ |  |
|  |  |  |
| **HIV RNA (copies/ml)** | **Monthly decline in CD4 cell count^2^** | **HIV RNA Distribution after Primary Infection^3^** |
| 0-20 | 3.0 | 0.00 |
| 20-500 | 3.0 | 0.012 |
| 501-3,000 | 3.7 | 0.023 |
| 3,001-10,000 | 4.6 | 0.078 |
| 10,001-30,000 | 5.4 | 0.18 |
| 30,001-100,000 | 6.4 | 0.28 |
| >100,000 | 6.4 | 0.43 |

1 Data from Granich et al. [[1](#_ENREF_1),[2](#_ENREF_2)]

2 Data from Mellors et al. [[3](#_ENREF_3)] and the Multicenter AIDS Cohort Study [[4](#_ENREF_4)].

3 Data from Lawn et al. [[5](#_ENREF_5)] and personal communication with Robin Wood [[6](#_ENREF_6)]

**References:**

1. Granich RM, Gilks CF, Dye C, De Cock KM, Williams BG (2009) Universal voluntary HIV testing with immediate antiretroviral therapy as a strategy for elimination of HIV transmission: a mathematical model. The Lancet 373: 48-57.

2. Williams BG, Korenromp EL, Gouws E, Schmid GP, Auvert B, et al. (2006) HIV infection, antiretroviral therapy, and CD4+ cell count distributions in African populations. The Journal of Infectious Diseases 194: 1450-1458.

3. Mellors JW, Muñoz A, Giorgi JV, Margolick JB, Tassoni CJ, et al. (1997) Plasma viral load and CD4+ lymphocytes as prognostic markers of HIV-1 infection. Annals of Internal Medicine 126: 946-954.

4. National Technical Information Service (2004) Multicenter AIDS cohort study (MACS) public dataset: release P12. Springfield, VA: National Technical Information Service.

5. Lawn SD, Badri M, Wood R (2005) Tuberculosis among HIV-infected patients receiving HAART: long term incidence and risk factors in a South African cohort. AIDS 19: 2109-2116.

6. Wood R (2006) Personal Communication with Robin Wood.
