# Supplementary material for: Development, Calibration and Performance of an HIV Transmission Model Incorporating Natural History and Behavioral Patterns: Application in South Africa
Source: PLoS One. 2014 May 27;9(5):e98272. doi: 10.1371/journal.pone.0098272 (PMC4035281; doi:10.1371/journal.pone.0098272)
Supplement: Table S3 — Parameters for HIV Prevalence Calibration. (DOCX) [file pone.0098272.s009.docx]

**Table S3: Parameters for HIV Prevalence Calibration**

| **Calendar Year** | **HIV Prevalence*** | **Low Estimate*** | **High Estimate*** |
| --- | --- | --- | --- |
| 1990 | 0.005 | 0.004 | 0.007 |
| 1991 | 0.008 | 0.006 | 0.01 |
| 1992 | 0.013 | 0.01 | 0.016 |
| 1993 | 0.021 | 0.018 | 0.024 |
| 1994 | 0.033 | 0.029 | 0.036 |
| 1995 | 0.049 | 0.044 | 0.053 |
| 1996 | 0.069 | 0.064 | 0.075 |
| 1997 | 0.092 | 0.085 | 0.099 |
| 1998 | 0.114 | 0.106 | 0.123 |
| 1999 | 0.113 | 0.124 | 0.142 |
| 2000 | 0.148 | 0.14 | 0.157 |
| 2001 | 0.159 | 0.151 | 0.168 |
| 2002 | 0.166 | 0.158 | 0.174 |

*****All prevalence data and high and low estimates were from UNAIDS 2010 data [[1](#_ENREF_1)]

**References:**

1. UNAIDS (2012) AIDSinfo Online Database - South Africa.
