# Supplementary material for: Development, Calibration and Performance of an HIV Transmission Model Incorporating Natural History and Behavioral Patterns: Application in South Africa
Source: PLoS One. 2014 May 27;9(5):e98272. doi: 10.1371/journal.pone.0098272 (PMC4035281; doi:10.1371/journal.pone.0098272)
Supplement: Table S4 — Parameters for Partnership Calibration. (DOCX) [file pone.0098272.s010.docx]

**Table S4: Parameters for Partnership Calibration**

| **Behavioral Restriction** | **Prior Range** | **Source** | **Posterior Weighted Mean** |
| --- | --- | --- | --- |
| Model Initialization Period | 600 months | MA |  |
| Proportion of the sexually active population in a steady relationship in the last year of prevalence delay | (0.23-0.39) | [[1-3](#_ENREF_1)] | 0.32 |
| Proportion of sexually active males in a casual partnership in the last year of prevalence delay | (0.15-0.60) | [[4-6](#_ENREF_4)] | 0.43 |
| Ratio of sexually active females to males in a casual partnership in the last year of prevalence delay | (0-1) | MA | 0.85 |
| Proportion of sexually active males in a CSW partnership in the last year of prevalence delay | (0.07-0.50) | MA, [[6](#_ENREF_6),[7](#_ENREF_7)] | 0.44 |
| Proportion of sexually active males with multiple partners in the last year of prevalence delay | (0.17-0.60) | [[6](#_ENREF_6),[8-12](#_ENREF_8)] | 0.47 |
| Ratio of sexually active females to males with multiple partners in the last year of prevalence delay | (0-1) | MA | 0.74 |
| Ratio of number of sex acts among sexually active low risk females to high risk females in the last month of prevalence delay | (0-1) | MA | 0.40 |
| Average number of sex acts per sexually active person in the last month of prevalence delay | (0-50) | [[13-16](#_ENREF_13)] | 8.86 |

**Abbreviation in the table:** MA= modeling assumption

**References:**

1. Statistics South Africa (1991) 1991 South Africa census.

2. Statistics South Africa (1996) 1996 South Africa census.

3. Statistics South Africa (2001) 2001 South Africa census.

4. Williams BG, Taljaard D, Campbell CM, Gouws E, Ndhlovu L, et al. (2003) Changing patterns of knowledge, reported behaviour and sexually transmitted infections in a South African gold mining community. AIDS 17: 2099-2107.

5. Harrison A, Cleland J, Frohlich J (2008) Young people's sexual partnerships in KwaZulu-Natal, South Africa: patterns, contextual influences, and HIV risk. Studies in Family Planning 39: 295-308.

6. Jewkes R, Nduna M, Levin J, Jama N, Dunkle K, et al. (2008) Impact of stepping stones on incidence of HIV and HSV-2 and sexual behaviour in rural South Africa: cluster randomised controlled trial. British Medical Journal 337: a506.

7. Carael M, Slaymaker E, Lyerla R, Sarkar S (2006) Clients of sex workers in different regions of the world: hard to count. Sexually Transmitted Infections 82: iii26-33.

8. Meekers D (2000) Going underground and going after women: trends in sexual risk behaviour among gold miners in South Africa. International Journal of STD and AIDS 11: 21-26.

9. Hargreaves JR, Bonell CP, Morison LA, Kim JC, Phetla G, et al. (2007) Explaining continued high HIV prevalence in South Africa: socioeconomic factors, HIV incidence and sexual behaviour change among a rural cohort, 2001-2004. AIDS 21: S39-48.

10. Shisana O, Rehle T, Simbayi L, Parker W, Zuma K, et al. (2005) South African national HIV prevalence, HIV incidence, behaviour and communication survery, 2005. Cape Town: HSRC Press.

11. Steffenson A (2008) Toward a Better Understanding of HIV Risk Among Young South Africans: Risk Perceptions and the Risk of Concurrent Sexual Partnerships: Harvard University.

12. Pettifor AE, Rees HV, Steffenson AE, Hlongwa-Madikizela L, MacPhail C, et al. (2004) HIV and sexual behaviour among young South Africans: a national survey of 15-24 year olds. Johannesburg: Reproductive Health Research Unit, University of the Witwatersrand.

13. Karim QA, Karim SS, Soldan K, Zondi M (1995) Reducing the risk of HIV infection among South African sex workers: socioeconomic and gender barriers. American Journal of Public Health 85: 1521-1525.

14. van Loggerenberg F, Mlisana K, Williamson C, Auld SC, Morris L, et al. (2008) Establishing a cohort at high risk of HIV infection in South Africa: challenges and experiences of the CAPRISA 002 acute infection study. PLOS ONE 3: e1954.

15. Varga CA (1997) The condom conundrum: barriers to condom use among commercial sex workers in Durban, South Africa. African Journal of Reproductive Health 1: 74-88.

16. Dunkle KL, Beksinska ME, Rees VH, Ballard RC, Htun Y, et al. (2005) Risk factors for HIV infection among sex workers in Johannesburg, South Africa. International Journal of STD and AIDS 16: 256-261.
