# Supplementary material for: Development, Calibration and Performance of an HIV Transmission Model Incorporating Natural History and Behavioral Patterns: Application in South Africa
Source: PLoS One. 2014 May 27;9(5):e98272. doi: 10.1371/journal.pone.0098272 (PMC4035281; doi:10.1371/journal.pone.0098272)
Supplement: Table S5 — Summary of the Phase 3 Calibration bounds. (DOCX) [file pone.0098272.s011.docx]

**Table S5: Summary of the Phase 3 Calibration bounds**

| **Restriction** | **Range for Partnership Calibration** | **Source** | **Posterior Weighted Mean** |
| --- | --- | --- | --- |
| Prevalence of HIV among CSW in 2002 | (0.30-0.65) | MA, [[1](#_ENREF_1)] | 0.52 |
| Prevalence of HIV among HR Males in 2002 | (0.30-0.65) | MA | 0.54 |
| Prevalence of HIV among HR Females in 2002 | (0.30-0.65) | MA, [[2](#_ENREF_2)] | 0.47 |
| Prevalence of HIV among LR Males in 2002 | (0-0.25) | MA, [[3](#_ENREF_3)] | 0.075 |
| Prevalence of HIV among LR Females in 2002 | (0-0.25) | MA, [[3](#_ENREF_3)] | 0.15 |
| Average number of partners per CSW/month in the last month of 2002 | (15-210) | MA, [[2](#_ENREF_2),[4](#_ENREF_4)] | 26.61 |
| 2-year incidence value (prior to 2002) | (0.01-0.18) | MA, [[2](#_ENREF_2),[3](#_ENREF_3)] | 0.054 |

**Table abbreviations**: CSW= commercial sex worker, HR = high risk, LR= low risk, and MA=modeling assumption.

**References:**

1. Dunkle KL, Beksinska ME, Rees VH, Ballard RC, Htun Y, et al. (2005) Risk factors for HIV infection among sex workers in Johannesburg, South Africa. International Journal of STD and AIDS 16: 256-261.

2. van Loggerenberg F, Mlisana K, Williamson C, Auld SC, Morris L, et al. (2008) Establishing a cohort at high risk of HIV infection in South Africa: challenges and experiences of the CAPRISA 002 acute infection study. PLOS ONE 3: e1954.

3. Shisana O, Rehle T, Simbayi L, Zuma K, Jooste S, et al. (2009) South African national HIV prevalence, incidence, behaviour and communication survey, 2008: a turning tide among teenagers? Cape Town: HSRC Press.

4. Karim QA, Karim SS, Soldan K, Zondi M (1995) Reducing the risk of HIV infection among South African sex workers: socioeconomic and gender barriers. American Journal of Public Health 85: 1521-1525.
