# Supplementary material for: Development, Calibration and Performance of an HIV Transmission Model Incorporating Natural History and Behavioral Patterns: Application in South Africa
Source: PLoS One. 2014 May 27;9(5):e98272. doi: 10.1371/journal.pone.0098272 (PMC4035281; doi:10.1371/journal.pone.0098272)
Supplement: Figure S1 — Cascade of parameter sets through the three stages of calibration. Beginning with 264,225 randomly chosen parameter sets, each parameter set was run through the model and exposed to three sets of restrictions to determine the runs that were most realistic in terms of sexual behavior and HIV prevalence predictions. A total of 3,750 runs passed all calibration restrictions and will be used in all future model analyses. (PDF) [file pone.0098272.s012.pdf]

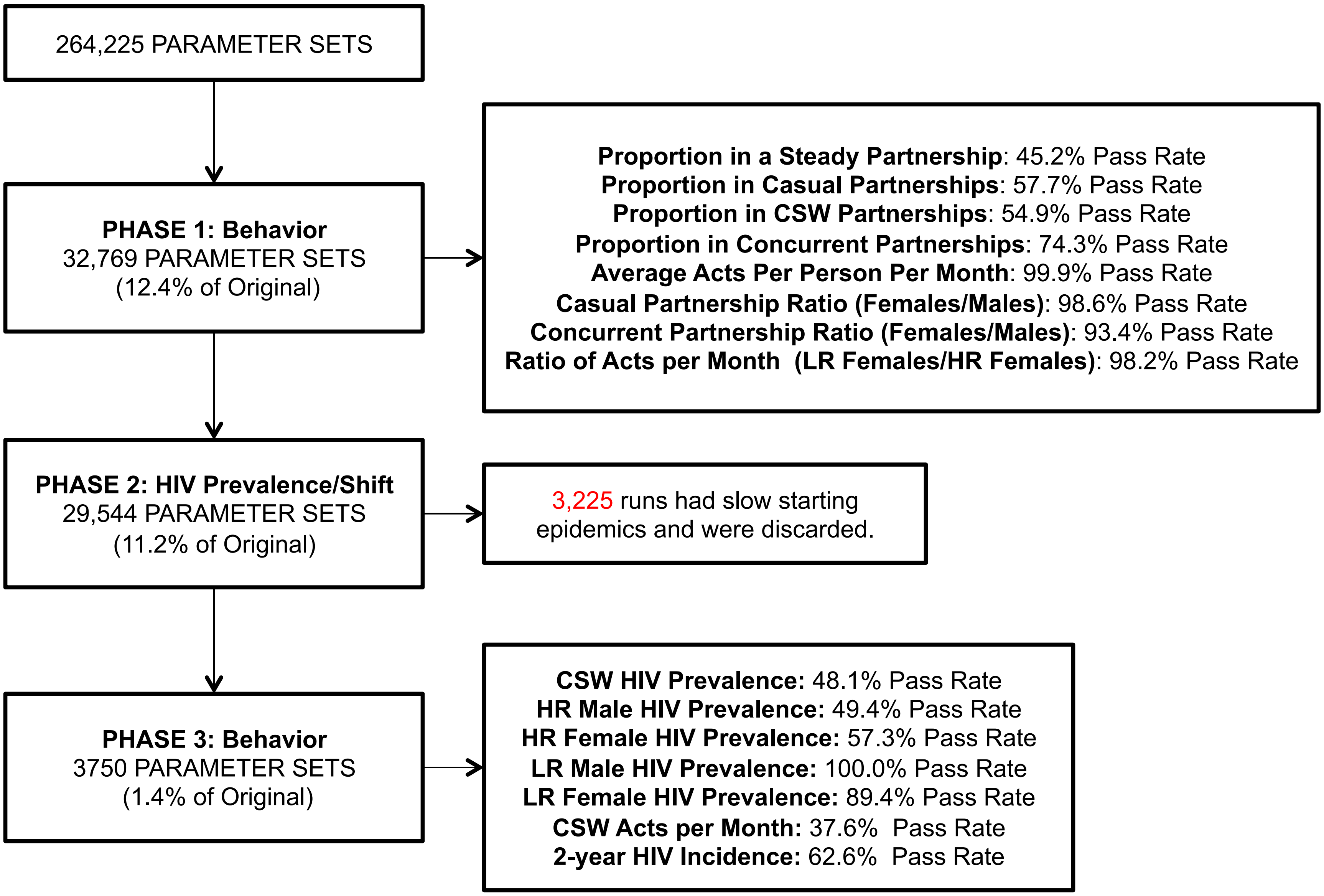
264,225 PARAMETER SETS
PHASE 1: Behavior
32,769 PARAMETER SETS
(12.4% of Original)
Proportion in a Steady Partnership: 45.2% Pass Rate
Proportion in Casual Partnerships: 57.7% Pass Rate
Proportion in CSW Partnerships: 54.9% Pass Rate
Proportion in Concurrent Partnerships: 74.3% Pass Rate
Average Acts Per Person Per Month: 99.9% Pass Rate
Casual Partnership Ratio (Females/Males): 98.6% Pass Rate
Concurrent Partnership Ratio (Females/Males): 93.4% Pass Rate
Ratio of Acts per Month (LR Females/HR Females): 98.2% Pass Rate
PHASE 2: HIV Prevalence/Shift
29,544 PARAMETER SETS
(11.2% of Original)
3,225 runs had slow starting epidemics and were discarded.
PHASE 3: Behavior
3750 PARAMETER SETS
(1.4% of Original)
CSW HIV Prevalence: 48.1% Pass Rate
HR Male HIV Prevalence: 49.4% Pass Rate
HR Female HIV Prevalence: 57.3% Pass Rate
LR Male HIV Prevalence: 100.0% Pass Rate
LR Female HIV Prevalence: 89.4% Pass Rate
CSW Acts per Month: 37.6% Pass Rate
2-year HIV Incidence: 62.6% Pass Rate
