# Supplementary figures and images for: Development, Calibration and Performance of an HIV Transmission Model Incorporating Natural History and Behavioral Patterns: Application in South Africa
Source: PLoS One. 2014 May 27;9(5):e98272. doi: 10.1371/journal.pone.0098272 (PMC4035281; doi:10.1371/journal.pone.0098272)

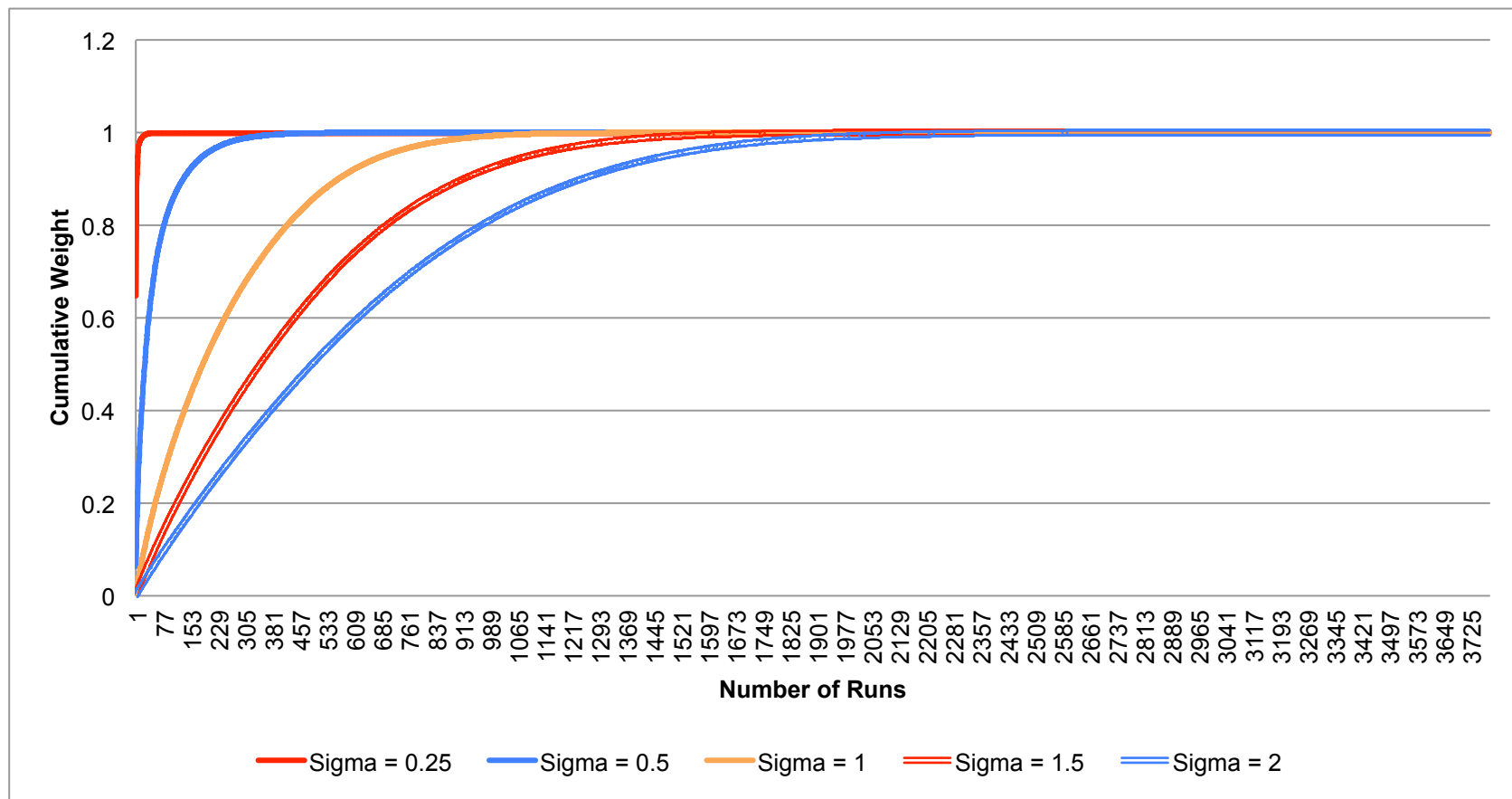

Supplement: Figure S2 — Cumulative runs vs. cumulative weight for runs passing the calibration procedure. This histogram depicts the cumulative distributions of weights (for a variety of sigma values) assigned to the 3,750 runs that passed the calibration procedure. The curve with sigma = 1 corresponds to the analyses described in the main text. The cut off (sigma = 1) for the 564 runs contributing to the top 90% of the weight is denoted by a red line. As noted from the figure, as sigma increases the number of runs that contribute to the top 90% of the weight to fit increases and the variance in the weights increases as well. (PDF) [file pone.0098272.s013.pdf]

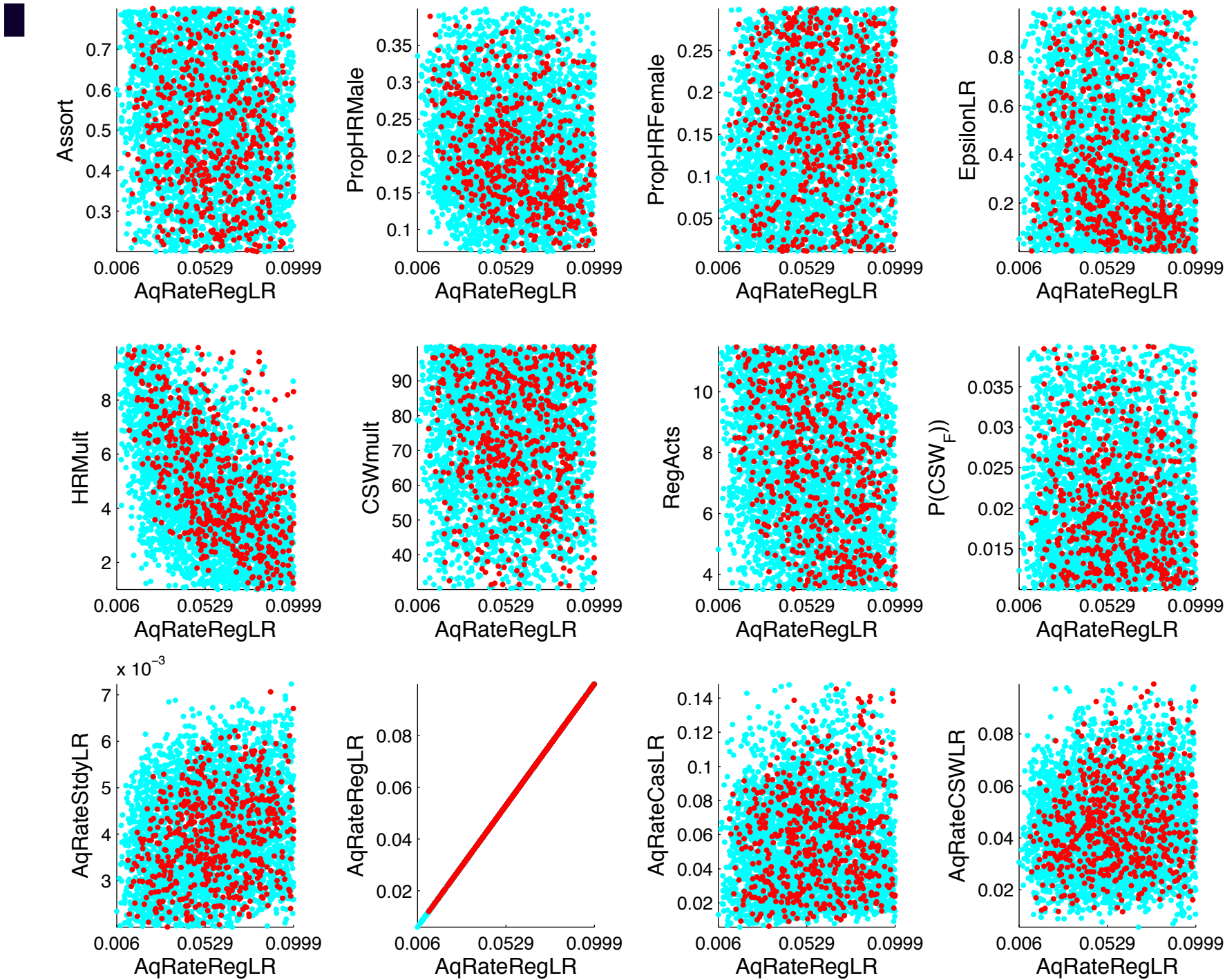

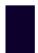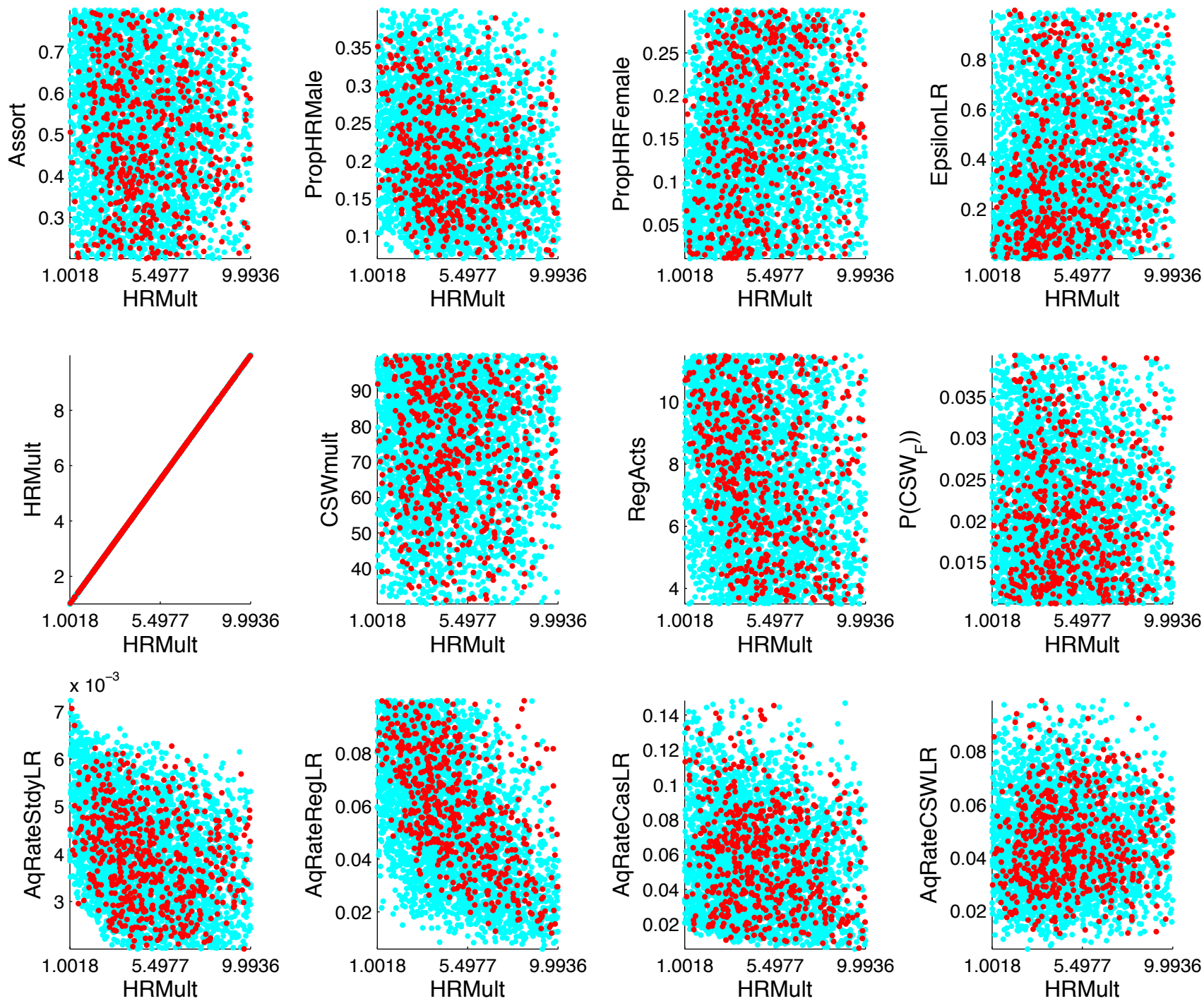

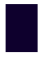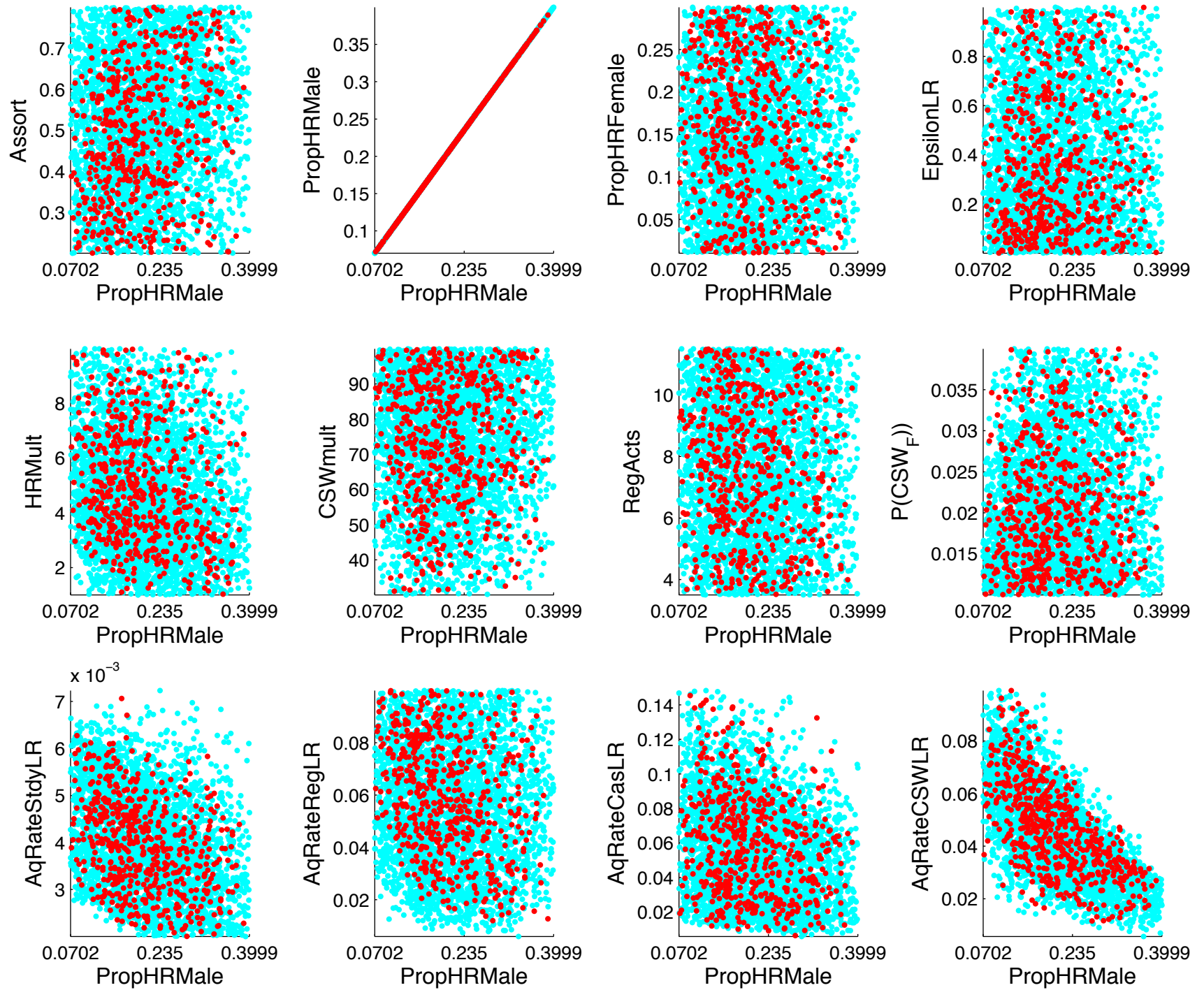

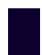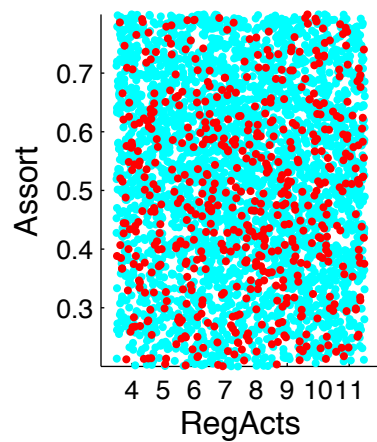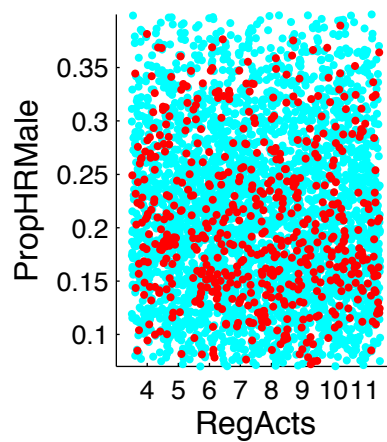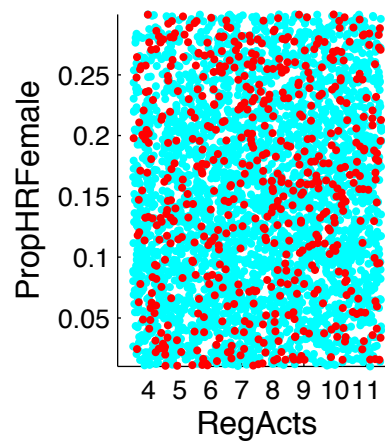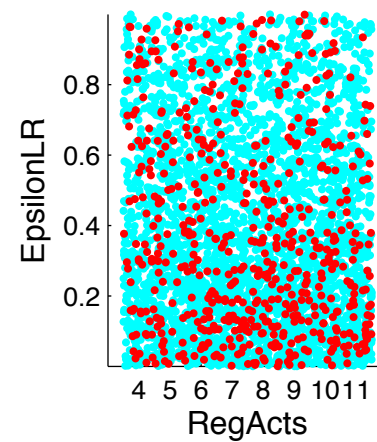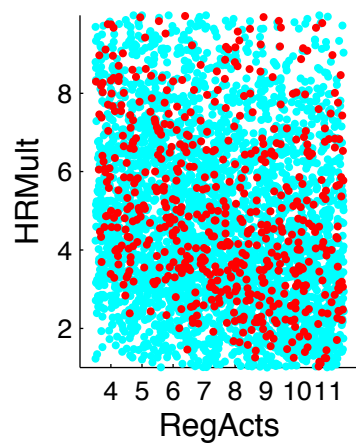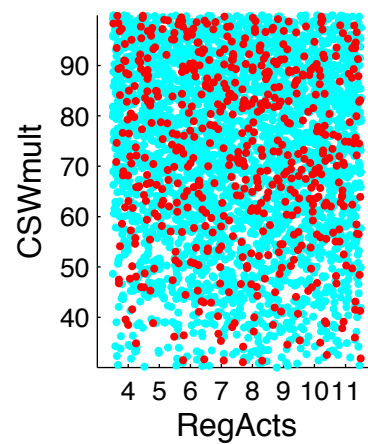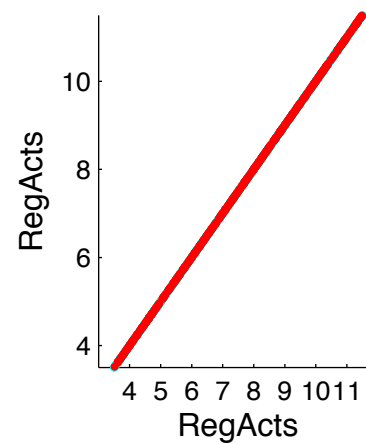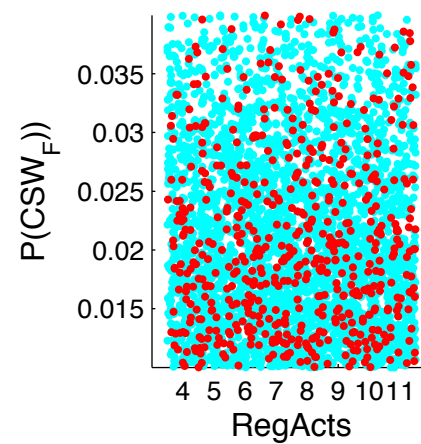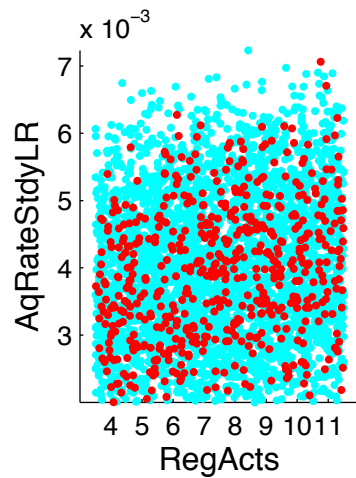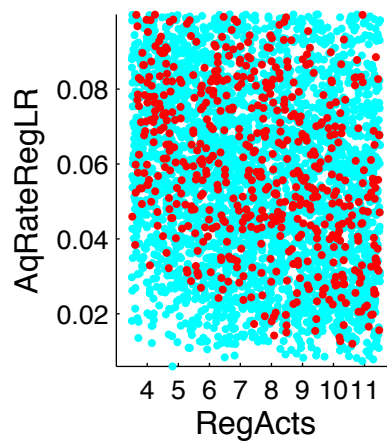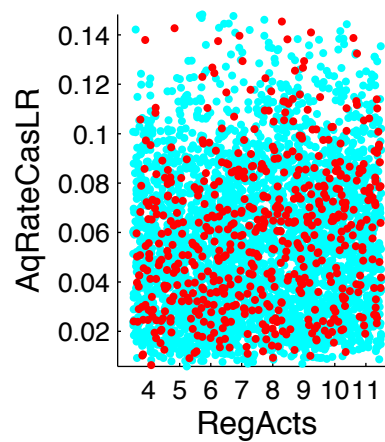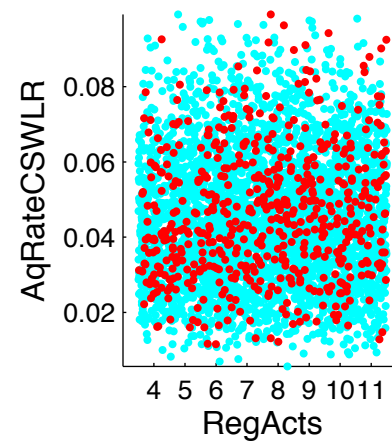

Supplement: Figure S4 — Two-dimensional plots of model fit. On each plot two CDM input parameters are varied, one on the x-axis and the other on the y-axis. A point was generated for each combination of the two parameter values that were part of one of the 3,750 parameter sets created in the fitting procedures. The color of each of these points represents the weight contribution for the plotted CDM model simulation with red representing parameter sets that contribute the top 90% of the weight and cyan representing parameter sets that contribute the bottom 10% of the weight. The plots represent the correlation between every parameter varied in the fitting procedures. The figure abbreviations are as follows: high-risk multiplier (HRMult), the CSW-risk multiplier (CSWmult), assortativeness parameter (Assort), proportion of males in the high-risk group (PropHRMale), proportion of females in the high-risk group (PropHRFemale), partner acquisition rate multiplier while in steady partnership (EpsilonLR), monthly partner acquisition rate for a steady partnership among low-risk males (AqRateStdyLR), monthly partner acquisition rate for a regular partnership among low-risk males (AqRateRegLR), monthly partner acquisition rate for a casual partnership among low-risk males (AqRateCasLR), monthly partner acquisition rate for a CSW encounter among low-risk males (AqRateCSWLR), chance of a female becoming a CSW (P(CSWF)), and number of acts per regular partnership per month among low and high-risk males (RegActs). The panels are as follows: A. The correlation between the monthly partner acquisition rate for a regular partnership among low-risk males (AqRateRegLR) and the 12 varied parameters; B. The correlation between the high-risk multiplier (HRMult) and the 12 varied parameters; C. The correlation between the proportion of males in the high-risk group (PropHRMale) and the 12 varied parameters; and D, The correlation between the number of acts per regular partnership per month among low and high-risk m [file pone.0098272.s015.pdf]

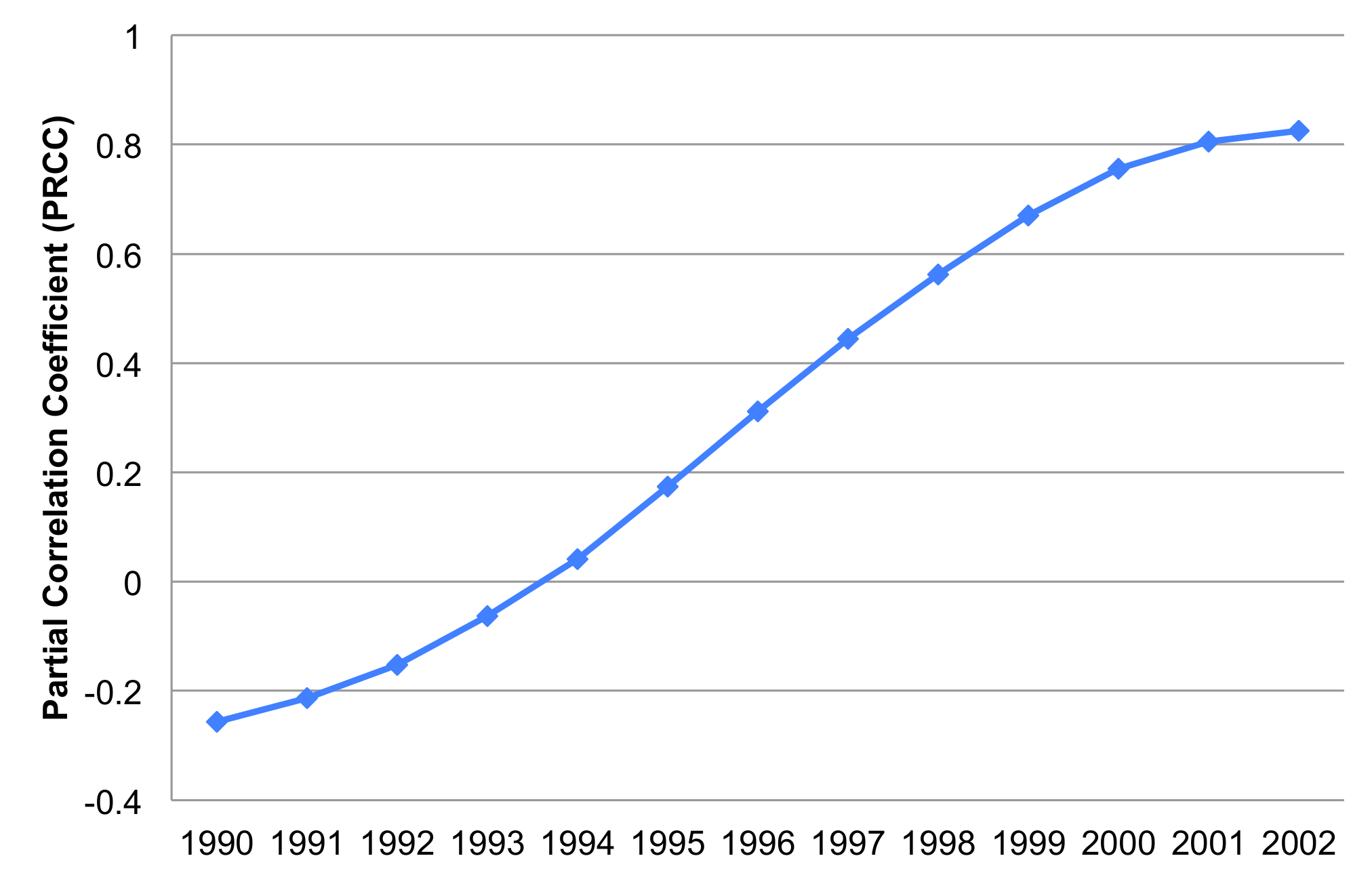

Supplement: Figure S5 — Partial rank correlation coefficients of concurrency in 1990 and HIV prevalence over time. This graph illustrates the relationship between the proportion of the population with greater than 2 partnerships in the last month of 1990 and the main outcome of interest, HIV prevalence in the sexually active population from 1990–2002. The partial rank correlation coefficient (PRCC) is plotted for each year between 1990–2002. The PRCC summarizes the impact of concurrency on the HIV prevalence in that year. A positive PRCC indicates that the HIV prevalence is positively correlated with concurrency (and inversely so for a negative PRCC). Despite a great deal of noise in the years prior to 1994, concurrency (defined as having 2 or more partners in the last month of 1990) positively influences the HIV prevalence for years after 1994. (TIFF) [file pone.0098272.s016.tiff]

A

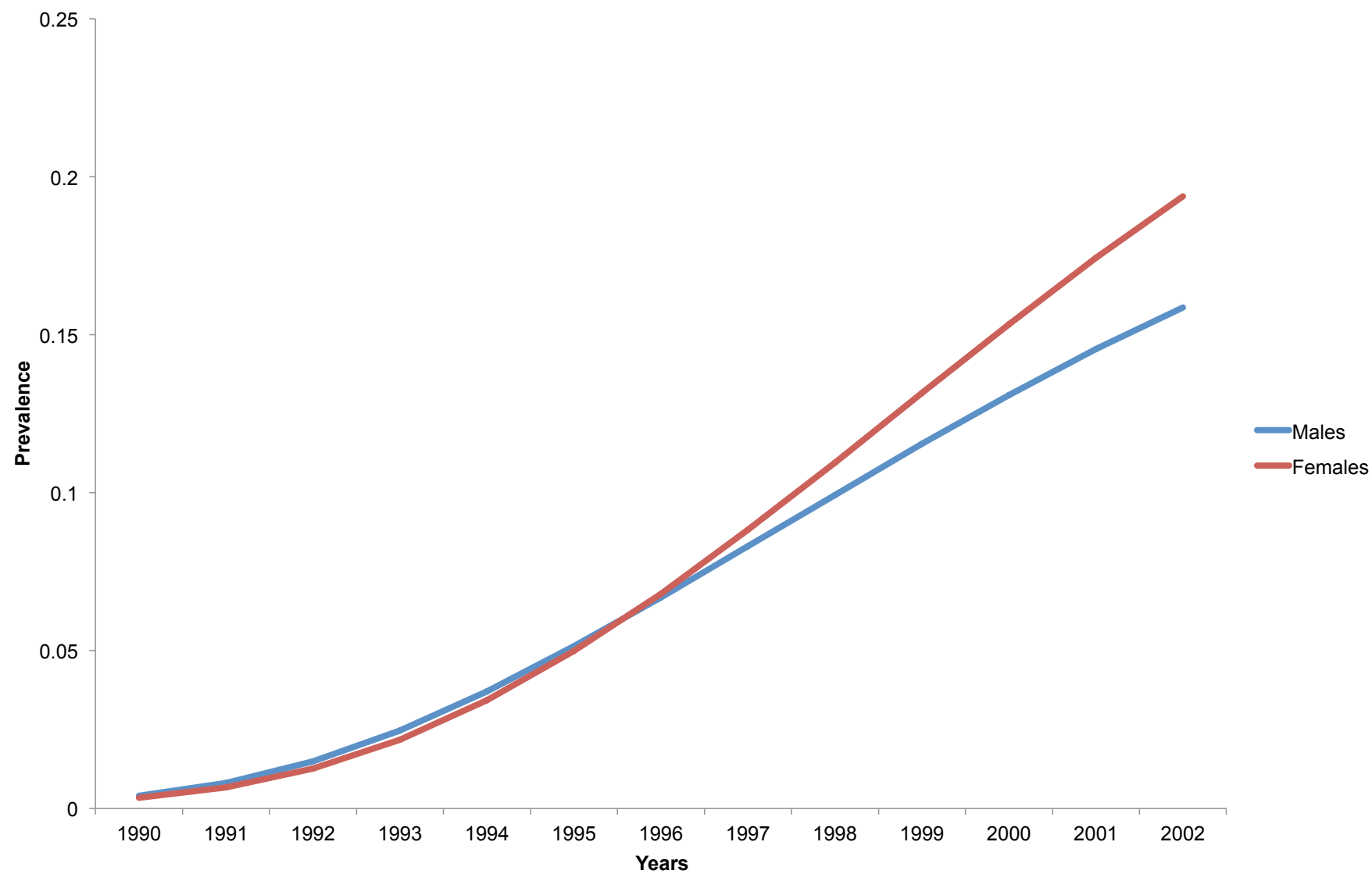

B

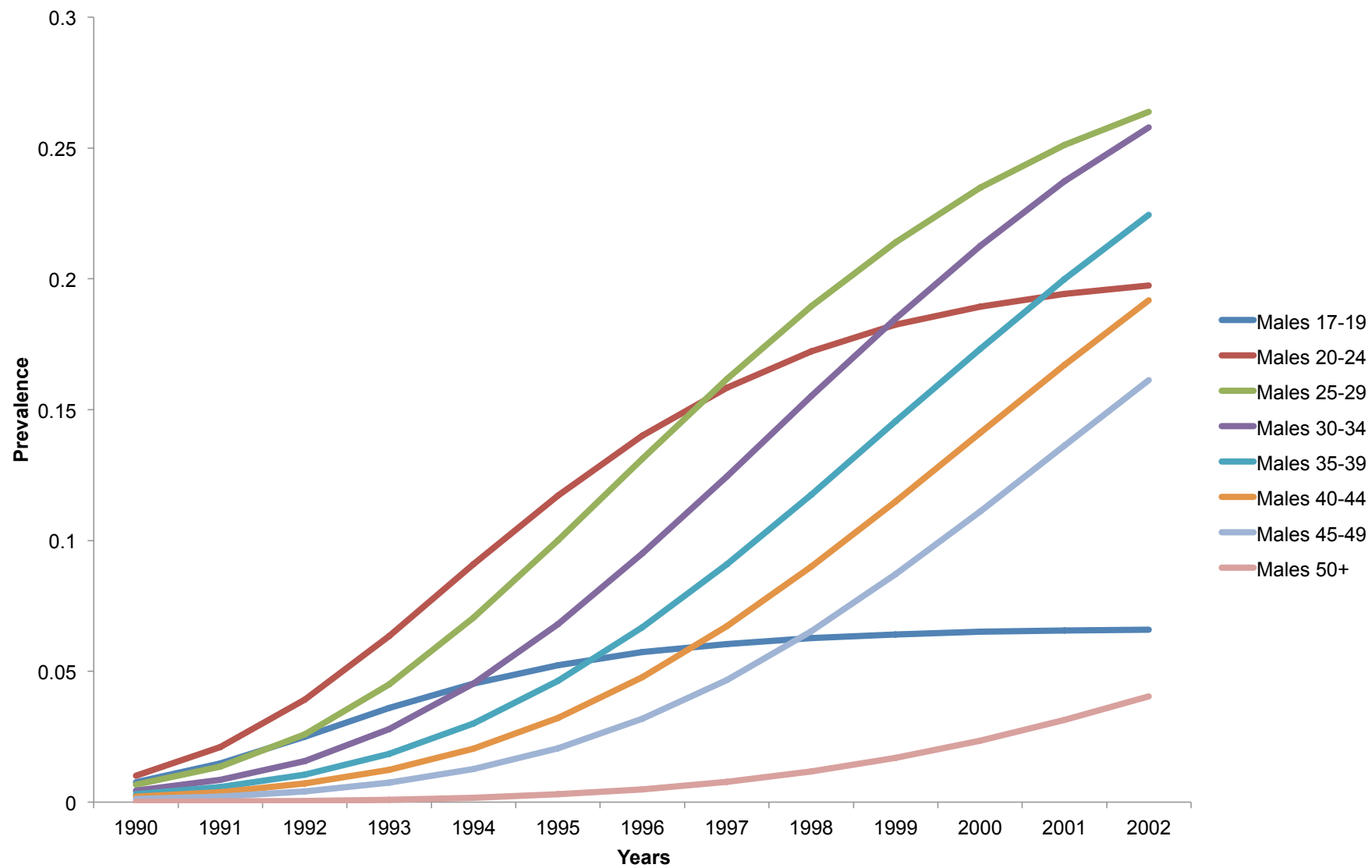

C

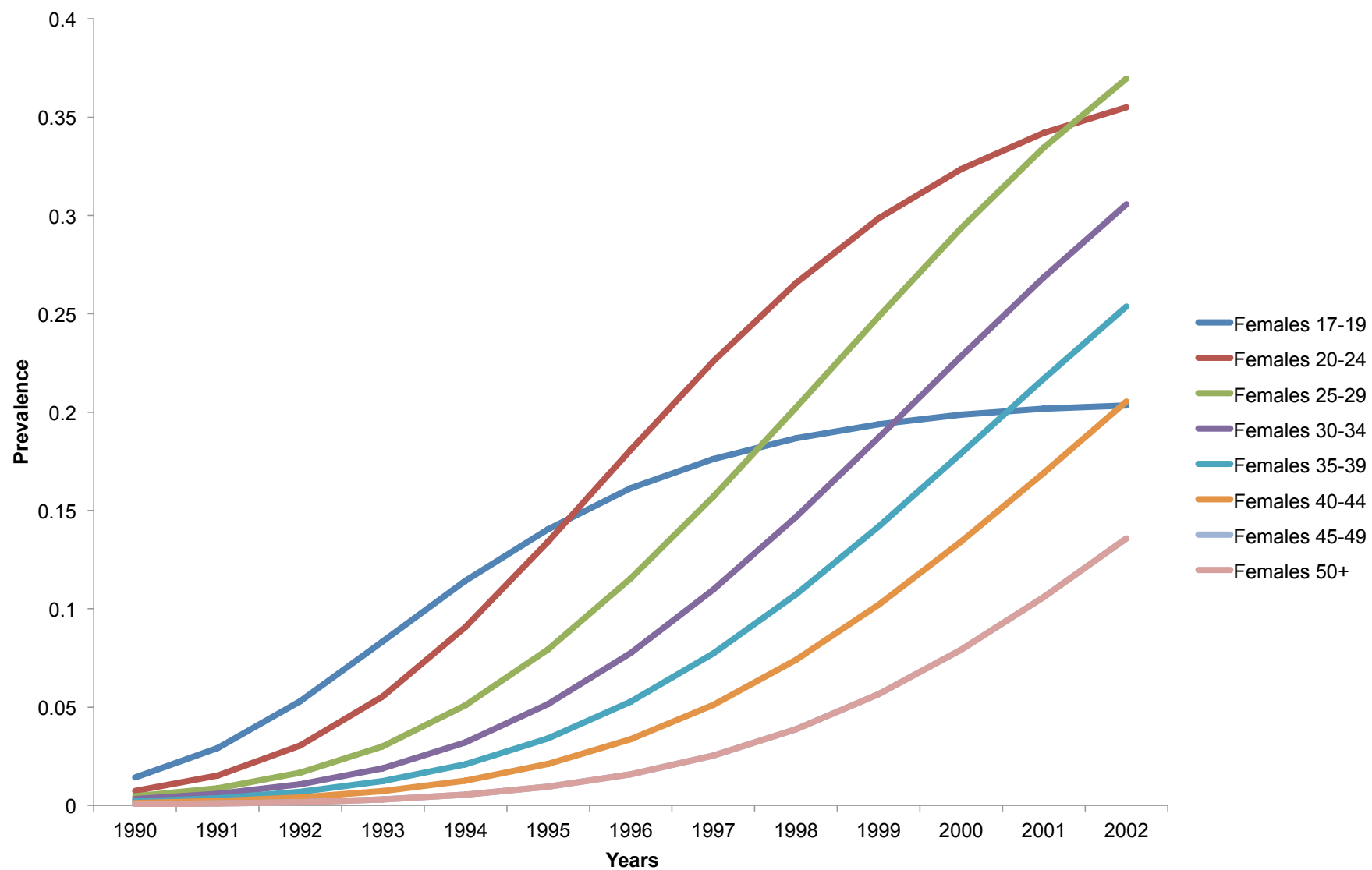

Supplement: Figure S6 — The CDM predicted HIV prevalence in South Africa from 1990–2002 stratified by age and gender. These graphs depict the weighted average of the HIV epidemic curves generated by the CDM, from the parameter sets that contribute the top 90% of the likelihood weight, stratified by gender and age. The panels are as follows: A. Depicts the weighted average HIV prevalence for males and females; B. Depicts the weighted average HIV prevalence for males stratified by the 8 age buckets modeled in the CDM; C. Depicts the weighted average HIV prevalence for females stratified by the 8 age buckets modeled in the CDM. (PDF) [file pone.0098272.s017.pdf]
