# Supplementary material for: Development, Calibration and Performance of an HIV Transmission Model Incorporating Natural History and Behavioral Patterns: Application in South Africa
Source: PLoS One. 2014 May 27;9(5):e98272. doi: 10.1371/journal.pone.0098272 (PMC4035281; doi:10.1371/journal.pone.0098272)

A. Assortativity

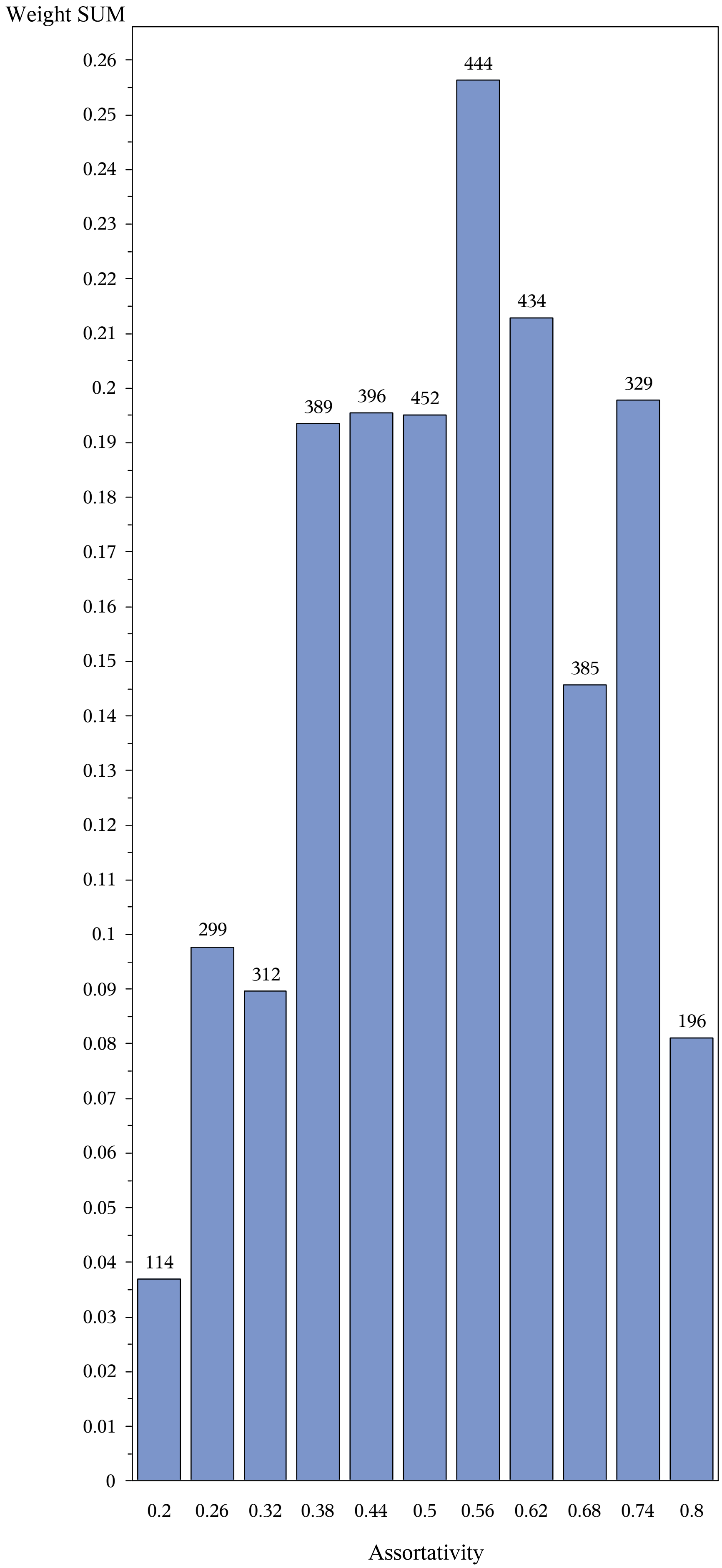

B. Number of Sexual Acts per Regular Partnership per Month

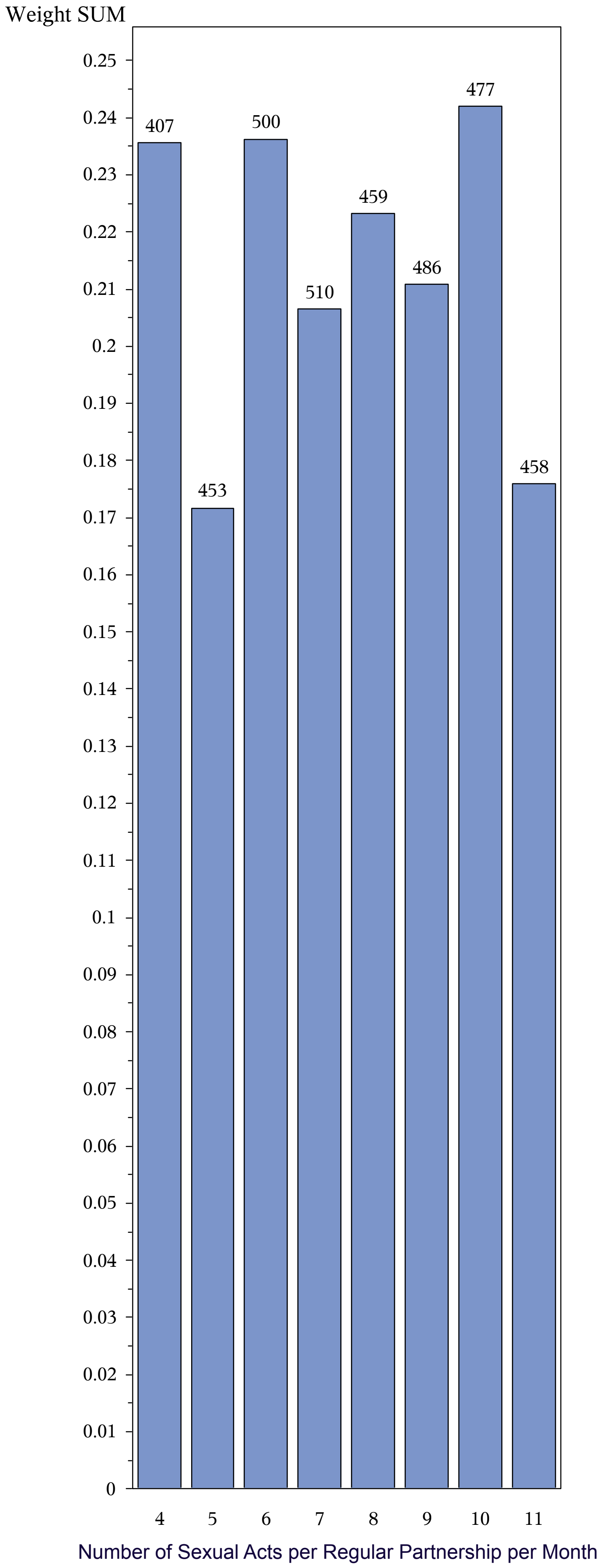

C. Chance of a Female Becoming a CSW

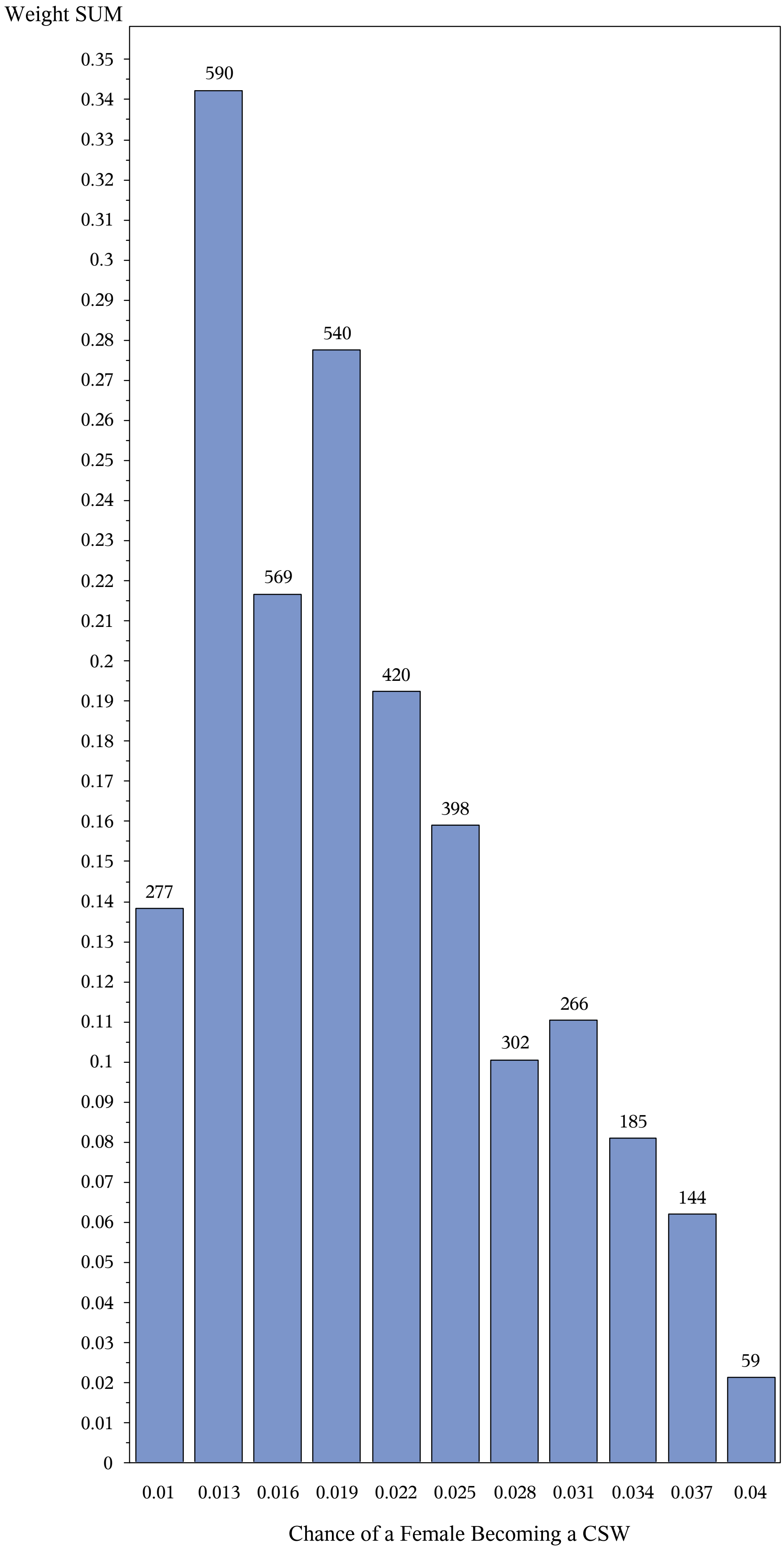

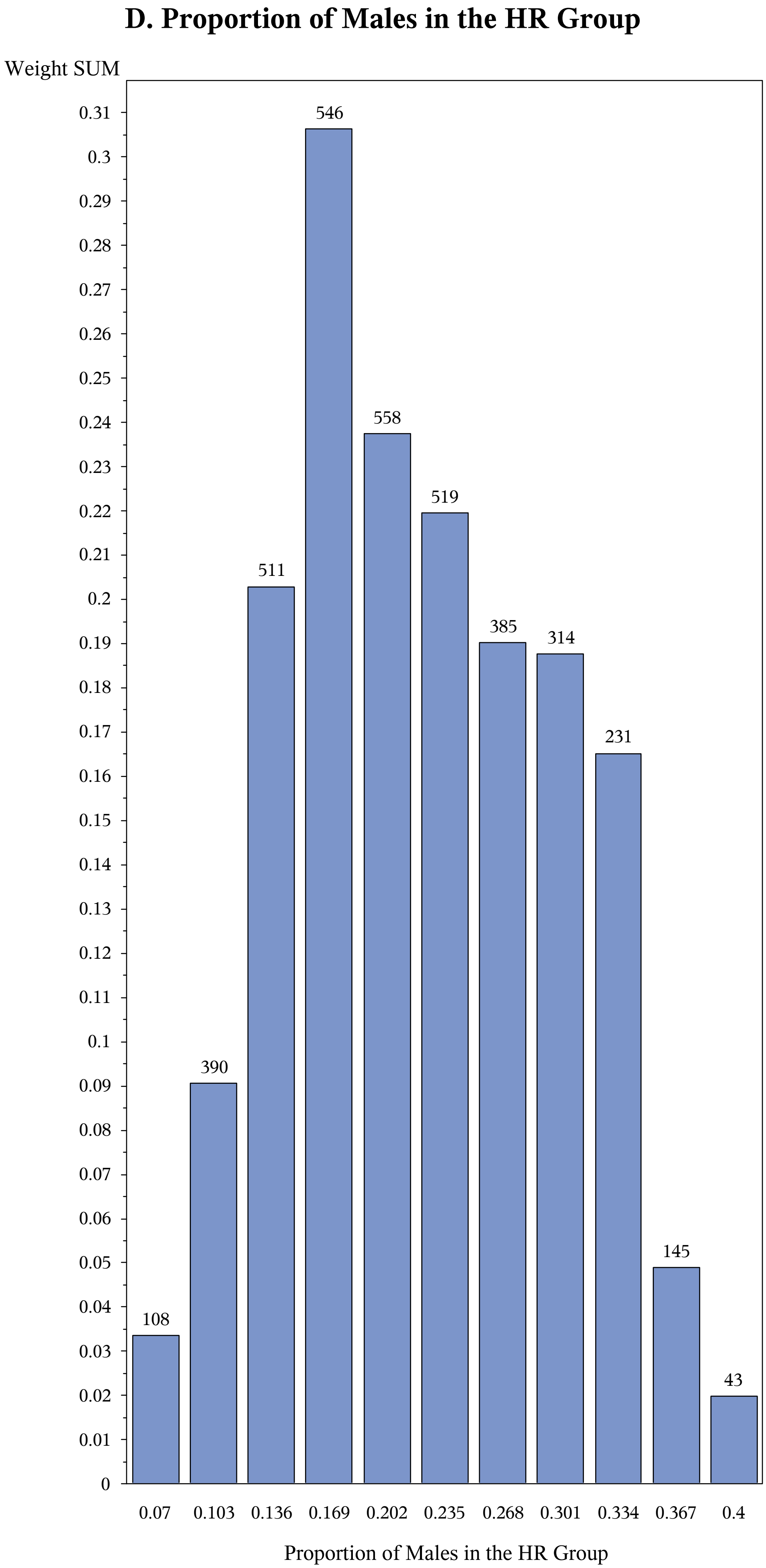
D. Proportion of Males in the HR Group
Weight SUM
0.31
0.3
0.29
0.28
0.27
0.26
0.25
0.24
0.23
0.22
0.21
0.2
0.19
0.18
0.17
0.16
0.15
0.14
0.13
0.12
0.11
0.1
0.09
0.08
0.07
0.06
0.05
0.04
0.03
0.02
0.01
0
108
390
511
546
558
519
385
314
231
145
43
0.07
0.103
0.136
0.169
0.202
0.235
0.268
0.301
0.334
0.367
0.4
Proportion of Males in the HR Group

E. Proportion of Non-CSW Females in the HR Group

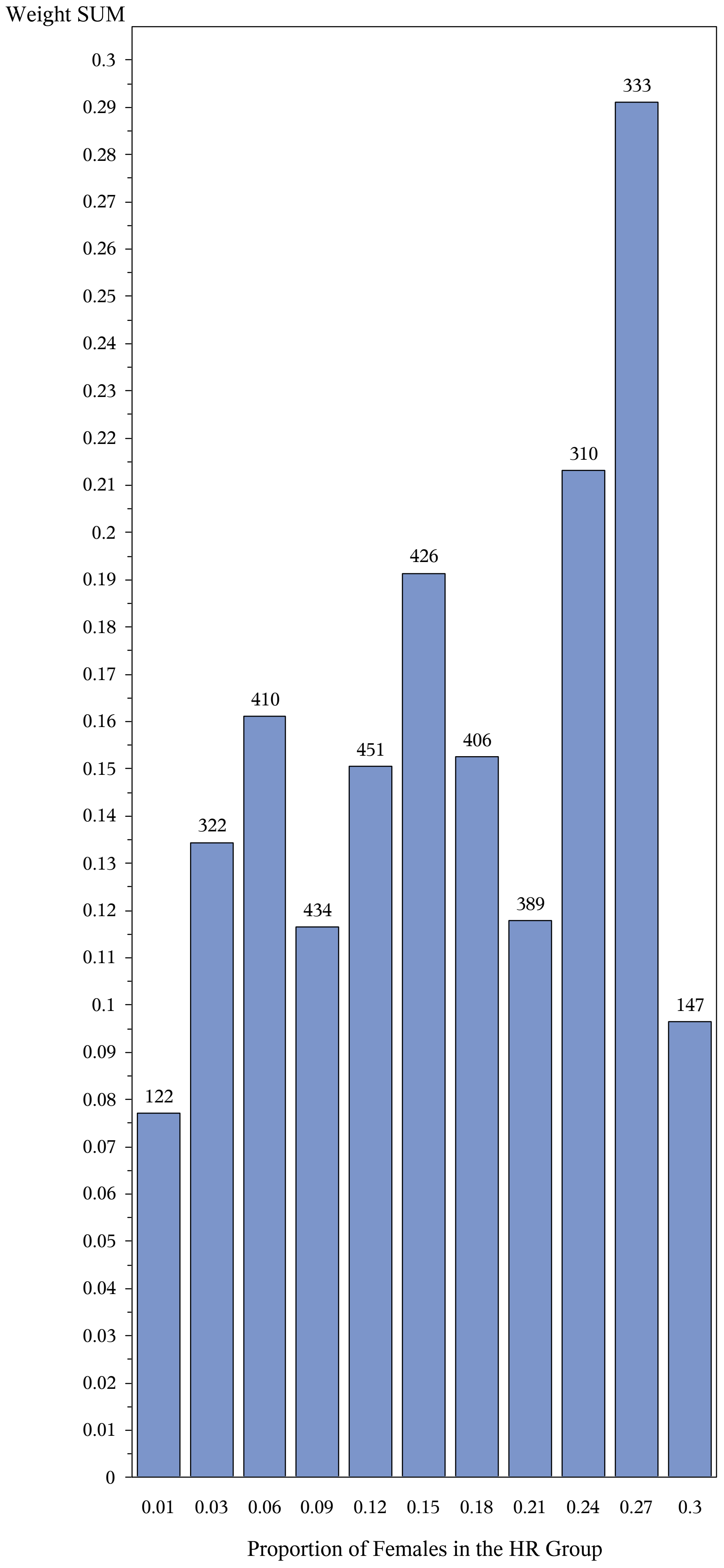

F. Partner Acquisition Rate Multiplier (Epsilon)

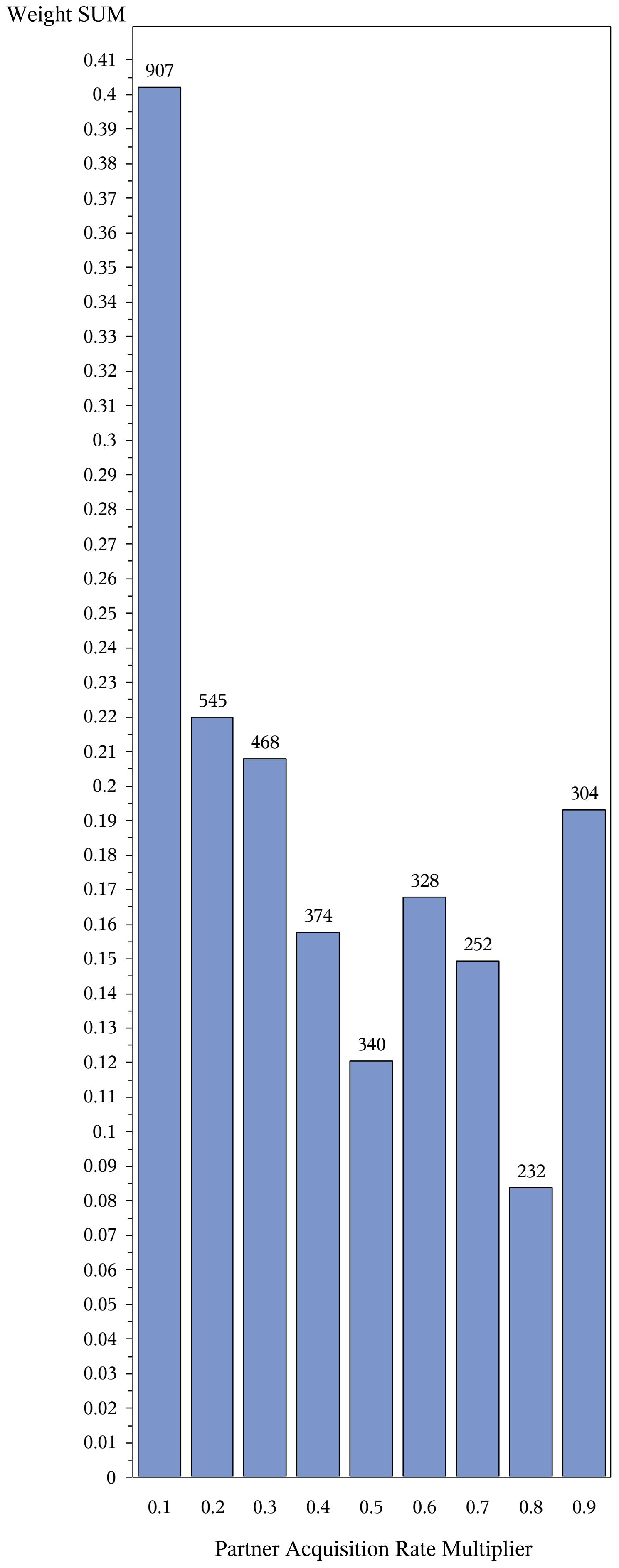

G. HR Multiplier

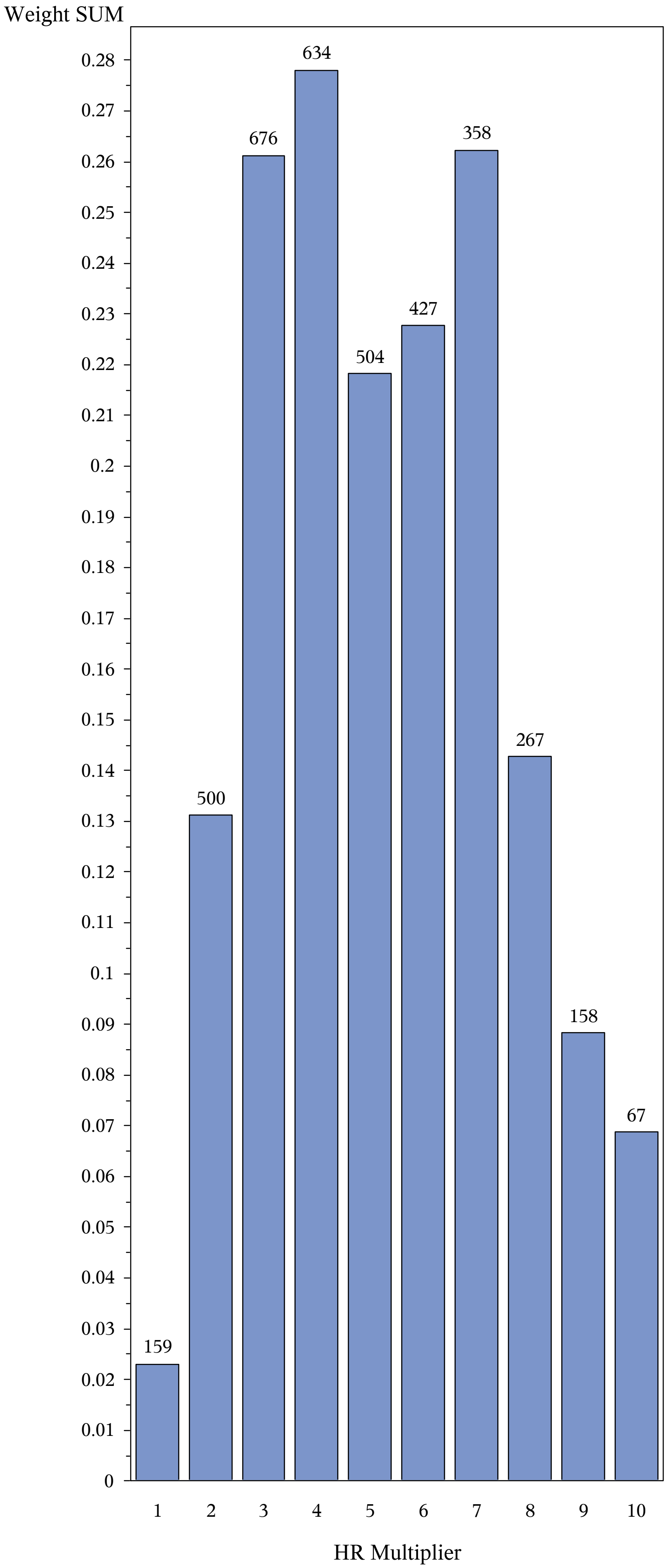

H. CSW Multiplier

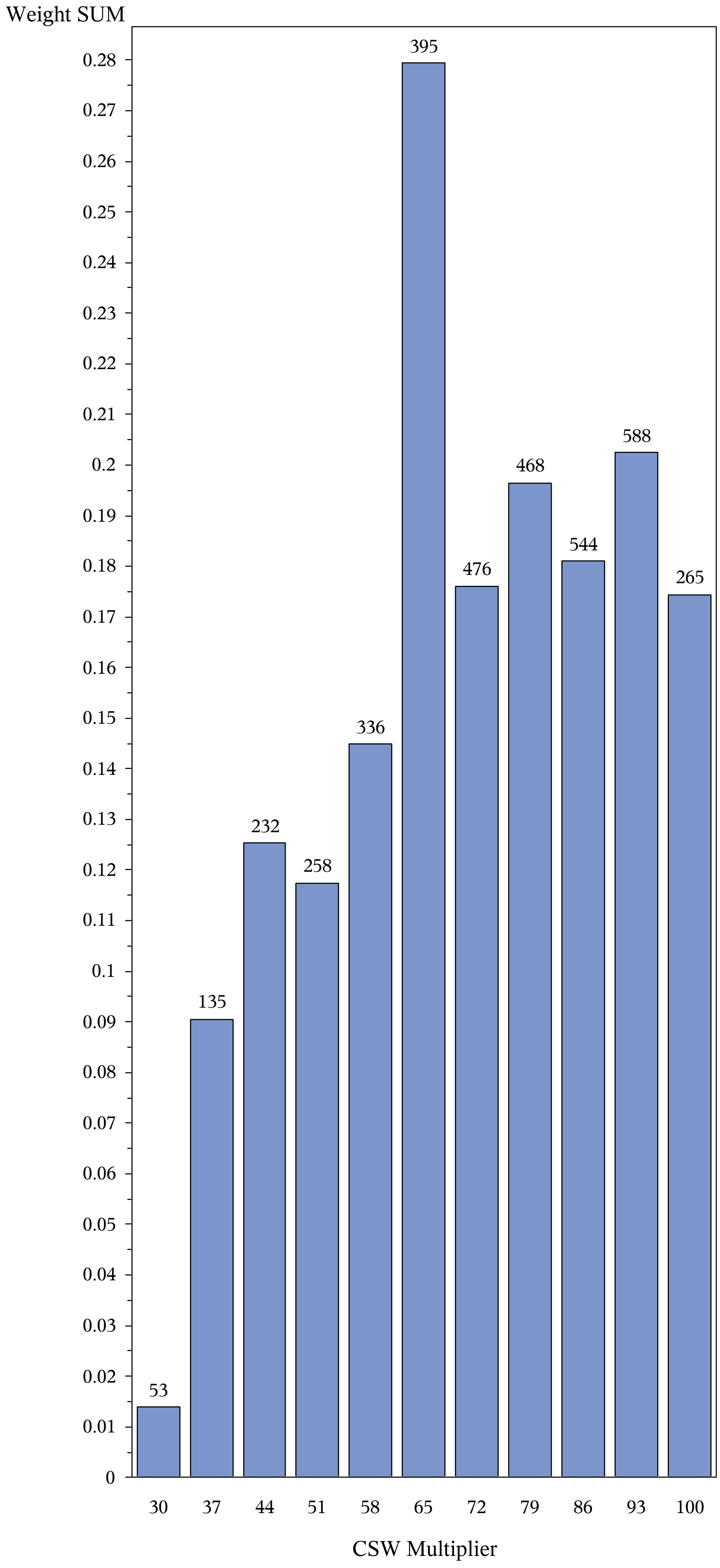

I. Monthly Acquisition Rate for Steady Partnerships for LR Males

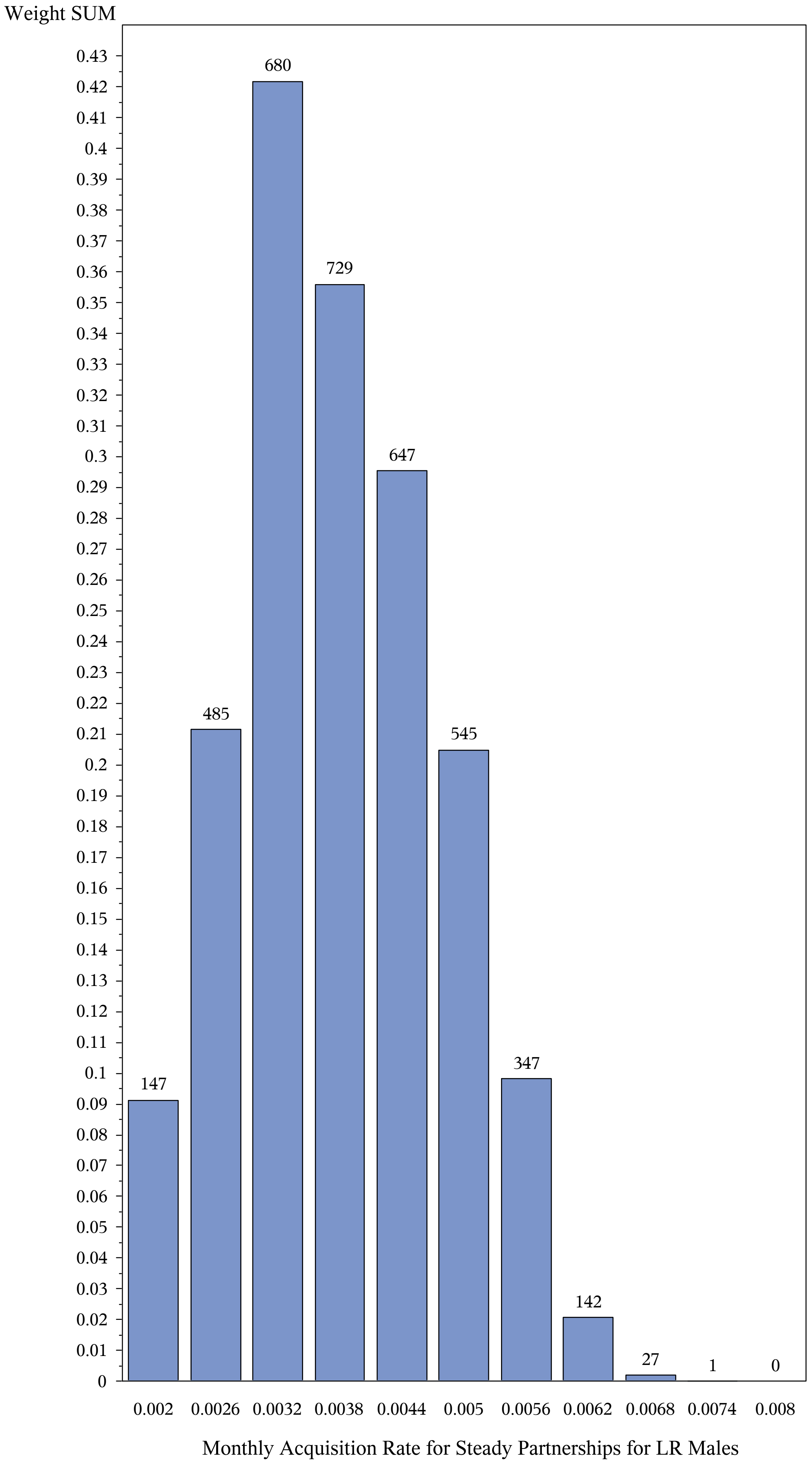

J. Monthly Acquisition Rate for Regular Partnerships for LR Males

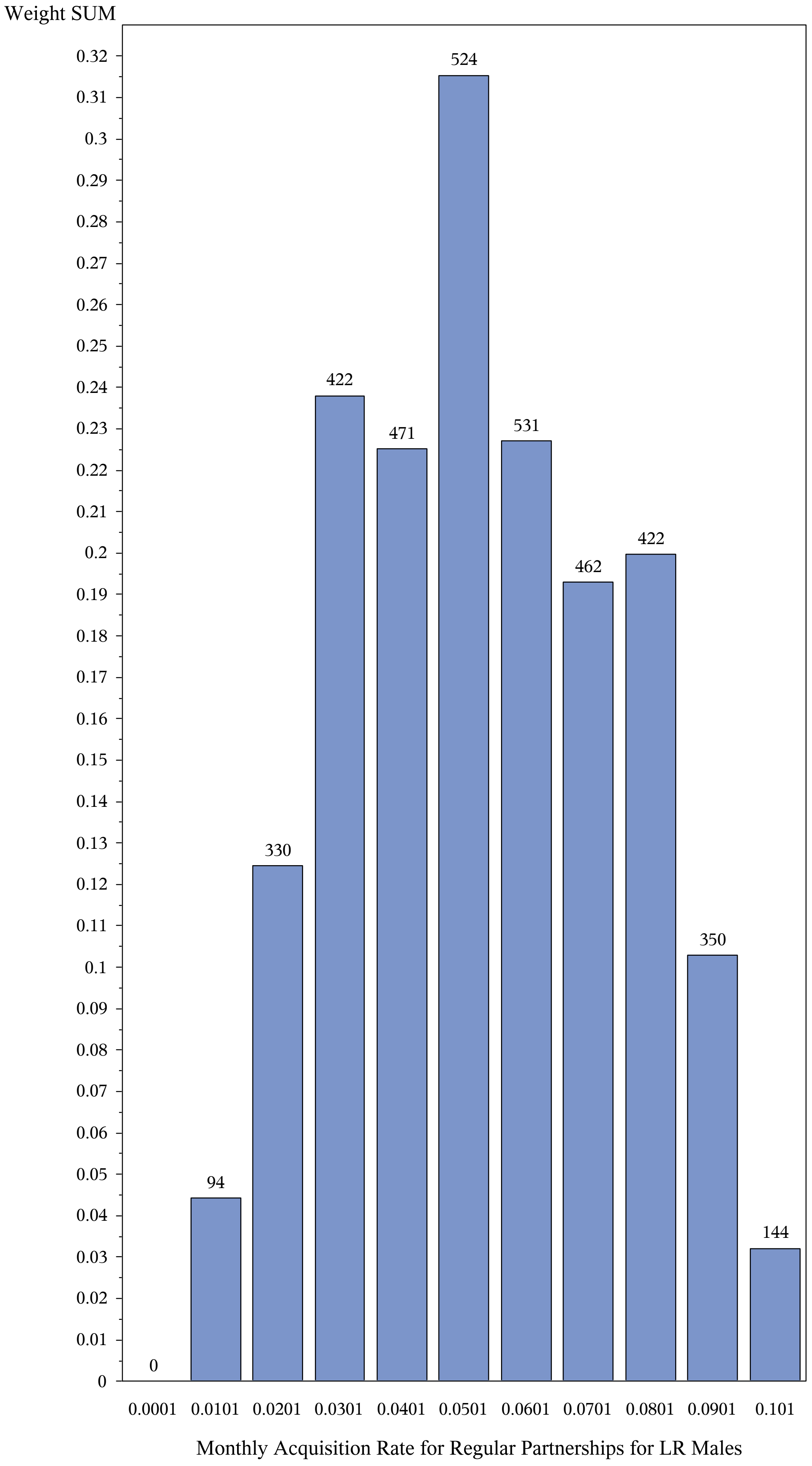

K. Monthly Acquisition Rate for Casual Partnerships for LR Males

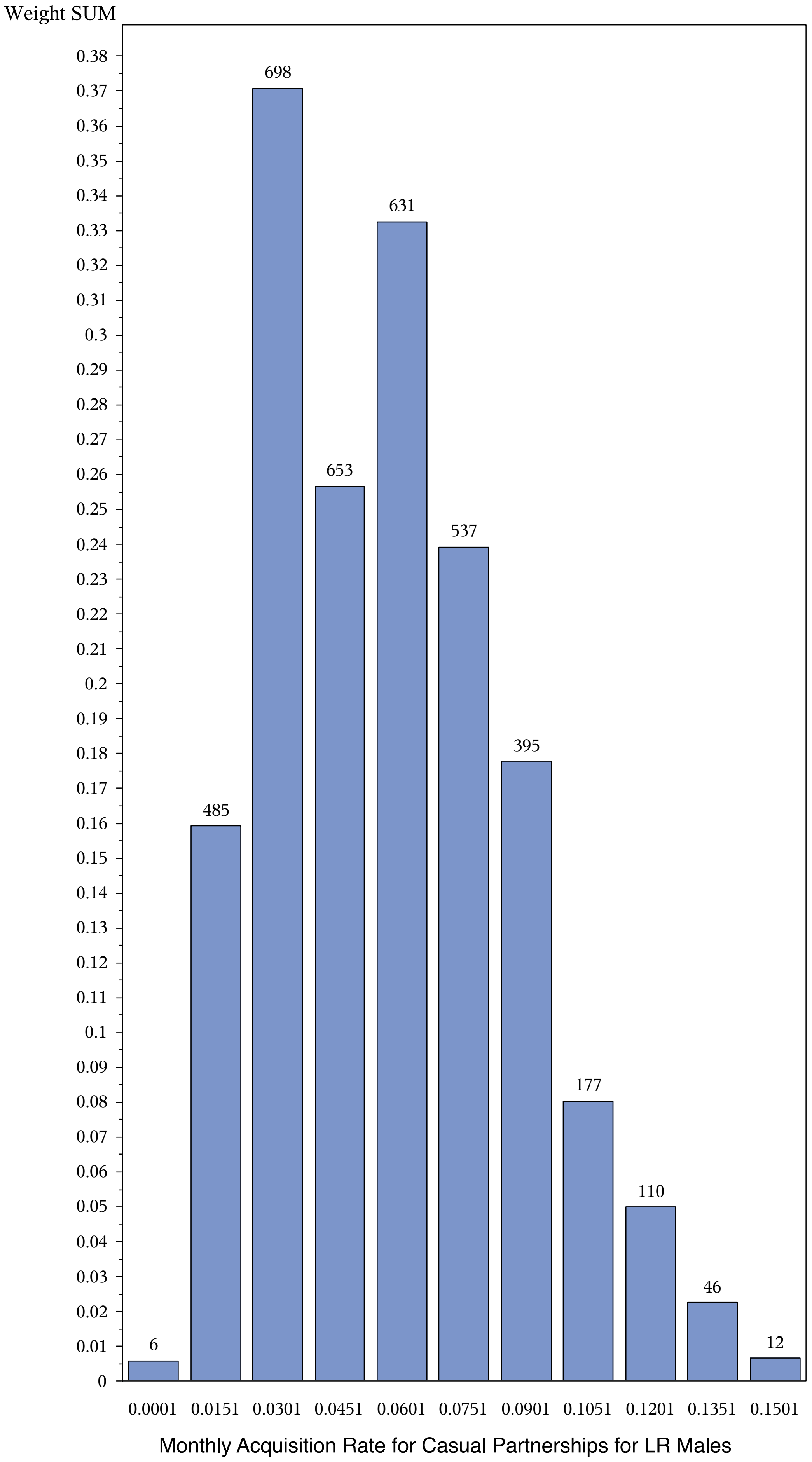

L. Monthly Acquisition Rate for CSW Partnerships for LR Males

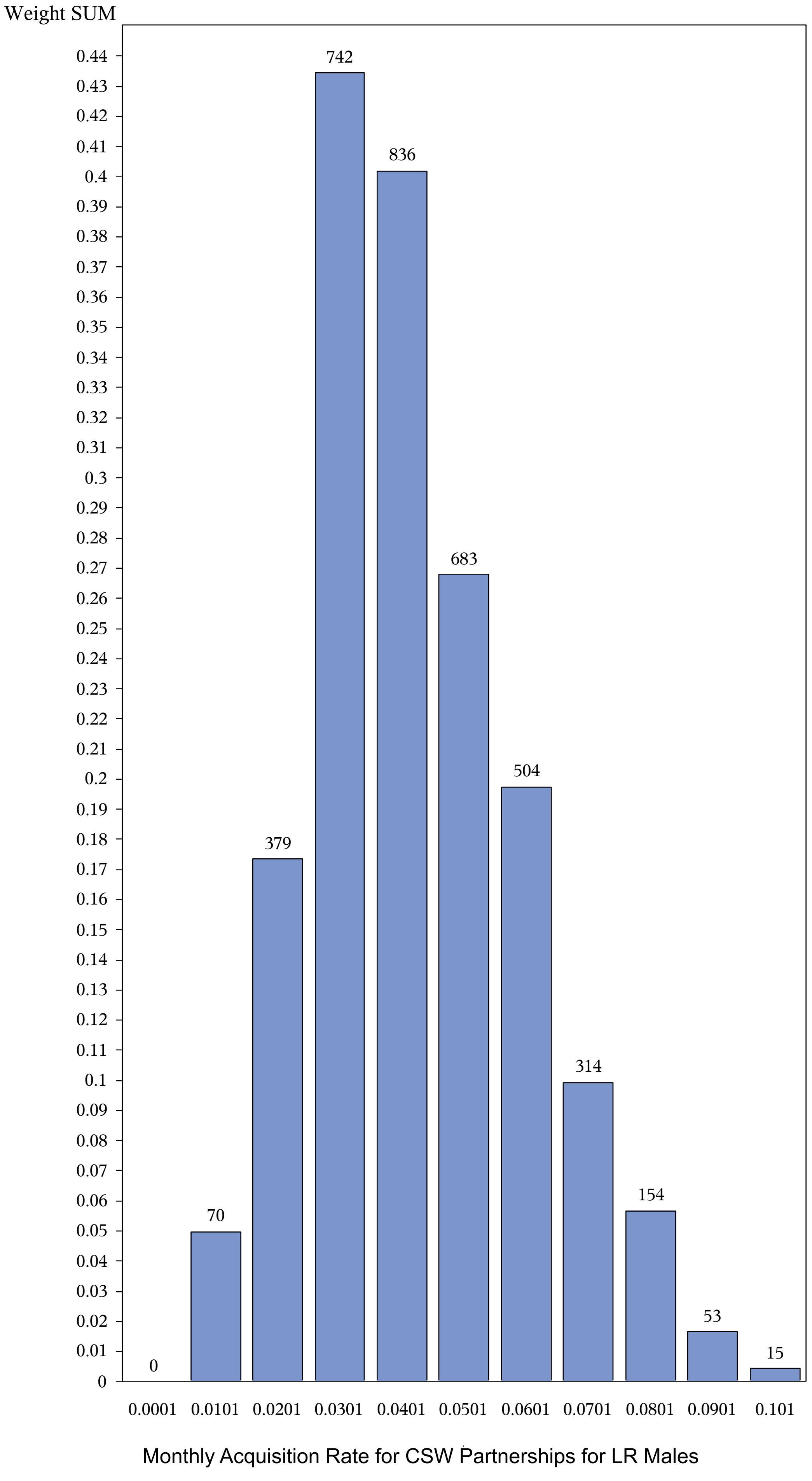

Supplement: Figure S3 — Posterior distributions for each parameter varied in the calibration procedure. Each histogram (A–L) represents the posterior distribution for each of the parameters varied in the calibration procedure with each of the bars representing the summed weight of the runs in that parameter value bin. The panels are as follows: A. Assortativity (Assort); B. Number of sexual acts per regular partnership per month (RegActs); C. Chance of a female becoming a CSW (ChanceCSW); D. Proportion of males in HR group (PropHRMale); E. Proportion of non-CSW females in HR group (PropHRFemale); F. Partnership acquisition rate multiplier (EpsilonLR); G. HR multiplier (HRMult); H. CSW multiplier (CSWMult); I. Monthly acquisition rate for steady partnerships for LR males (AqRateStdyLR); J. Monthly acquisition rate for regular partnerships for LR males (AqRateRegLR); K. Monthly acquisition rate for casual partnerships for LR males (AqRateCasLR); and L. Monthly acquisition rate for CSW partnerships for LR males (AqRateCSWLR). (PDF) [file pone.0098272.s014.pdf]
